# ATOMICA: Learning Universal Representations of Intermolecular Interactions

**DOI:** 10.1101/2025.04.02.646906

**Published:** 2025-04-08

**Authors:** Ada Fang, Zaixi Zhang, Andrew Zhou, Marinka Zitnik

## Abstract

Molecular interactions underlie nearly all biological processes, but most machine learning models treat molecules in isolation or specialize in a single type of interaction, such as protein-ligand or protein-protein binding. This siloed approach prevents generalization across biomolecular classes and limits the ability to model interaction interfaces systematically. We introduce ATOMICA, a geometric deep learning model that learns atomic-scale representations of intermolecular interfaces across diverse biomolecular modalities, including small molecules, metal ions, amino acids, and nucleic acids. ATOMICA uses a self-supervised denoising and masking objective to train on 2,037,972 interaction complexes and generate hierarchical embeddings at the levels of atoms, chemical blocks, and molecular interfaces. The model generalizes across molecular classes and recovers shared physicochemical features without supervision. Its latent space captures compositional and chemical similarities across interaction types and follows scaling laws that improve representation quality with increasing biomolecular data modalities. We apply ATOMICA to construct five modality-specific interfaceome networks, termed ATOMICAN et s, which connect proteins based on interaction similarity with ions, small molecules, nucleic acids, lipids, and proteins. These networks identify disease pathways across 27 conditions and predict disease-associated proteins in autoimmune neuropathies and lymphoma. Finally, we use ATOMICA to annotate the dark proteome—proteins lacking known structure or function—by predicting 2,646 previously uncharacterized ligand-binding sites. These include putative zinc finger motifs and transmembrane cytochrome subunits, demonstrating that ATOMICA enables systematic annotation of molecular interactions across the proteome.

## Full Text Availability

The license terms selected by the author(s) for this preprint version do not permit archiving in PMC. The full text is available from the preprint server.

