# Learning Universal Representations of Intermolecular Interactions with ATOMICA

**DOI:** 10.1101/2025.04.02.646906

**Published:** 2025-07-15

**Authors:** Ada Fang, Michael Desgagné, Zaixi Zhang, Andrew Zhou, Joseph Loscalzo, Bradley L. Pentelute, Marinka Zitnik

**Affiliations:** 1 Department of Chemistry and Chemical Biology, Harvard University, Cambridge, MA, USA; 2 Kempner Institute for the Study of Natural and Artificial Intelligence, Harvard University, MA, USA; 3 Department of Biomedical Informatics, Harvard Medical School, Boston, MA, USA; 4 Department of Chemistry, Massachusetts Institute of Technology, Cambridge, MA, USA; 5 Program in Health Sciences and Technology, Massachusetts Institute of Technology, Cambridge, USA; 6 Department of Medicine, Harvard Medical School, Boston, MA, USA; 7 Brigham and Women’s Hospital, Boston, MA, USA; 8 Koch Institute for Integrative Cancer Research, Massachusetts Institute of Technology, Cambridge, MA, USA; 9 Center for Environmental Health Sciences, Massachusetts Institute of Technology, Cambridge, MA, USA; 10 Broad Institute of MIT and Harvard, Cambridge, MA, USA; 11 Harvard Data Science Initiative, Cambridge, MA, USA

## Abstract

Molecular interactions underlie nearly all biological processes, but most machine learning models treat molecules in isolation or specialize in a single type of interaction, such as protein-ligand or protein-protein binding. Here, we introduce ATOMICA, a geometric deep learning model that learns atomic-scale representations of intermolecular interfaces across five modalities, including proteins, small molecules, metal ions, lipids, and nucleic acids. ATOMICA is trained on 2,037,972 interaction complexes using self-supervised denoising and masking to generate embeddings of interaction interfaces at the levels of atoms, chemical blocks, and molecular interfaces. ATOMICA’s latent space is compositional and captures physicochemical features shared across molecular classes, enabling representations of new molecular interactions to be generated by algebraically combining embeddings of interaction interfaces. The representation quality of this space improves with increased data volume and modality diversity. As in pre-trained natural language models, this scaling law implies predictable gains in performance as structural datasets expand. We construct modality-specific interfaceome networks, termed ATOMICANets, which connect proteins based on interaction similarity with ions, small molecules, nucleic acids, lipids, and proteins. By overlaying disease-associated proteins of 27 diseases onto ATOMICANets, we find strong associations for asthma in lipid networks and myeloid leukemia in ion networks. We use ATOMICA to annotate the dark proteome—proteins lacking known function—by predicting 2,646 uncharacterized ligand-binding sites, including putative zinc finger motifs and transmembrane cytochrome subunits. We experimentally confirm heme binding for five ATOMICA predictions in the dark proteome. By modeling molecular interactions, ATOMICA opens new avenues for understanding and annotating molecular function at scale.

## Main

**Figure 1:**
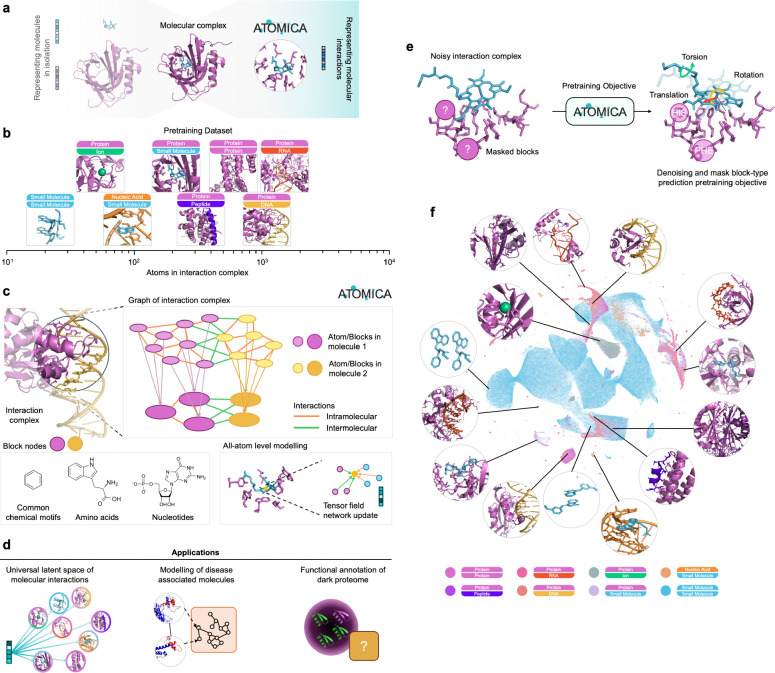
Overview of ATOMICA pretraining data, architecture, and latent space. **a** ATOMICA focuses on representing molecular interactions which is complementary to models representing individual molecules. **b** Input modalities and the number of atoms in the interaction complexes that ATOMICA are pretrained on. **c** Overview of ATOMICA architecture, interaction complexes are modeled at the atom and block level. Message passing between nodes at each level is done via intermolecular and intramolecular edges. **d** ATOMICA enables modeling of a universal latent space of intermolecular interactions. This is leveraged for the modeling of disease associated molecules and functional annotation of the dark proteome. **e** Self-supervised pretraining objectives for generating ATOMICA embeddings. **f** UMAP of latent space of all interaction complexes seen during pretraining.

Molecular interactions influence all aspects of chemistry and biology. Despite advances in structure prediction and molecular modeling, machine learning models model molecules in isolation or are restricted to a single type of molecular interaction (Fig. 1a). Protein and nucleic acid models leverage sequence-based tokenization [1–5], whereas small molecules require atomic-scale modeling due to their lack of inherent sequential structure [6–11]. Molecular interaction models lack generalizability across molecular modalities, with distinct architectures designed for protein-ligand binding affinity [12–16], binding site prediction [17–20], protein-peptide interactions [21–23], protein-protein interactions [24–29], and protein-RNA recognition [30–35]. This siloed approach limits the ability to transfer knowledge across molecular classes, even though interactions among proteins, nucleic acids, small molecules, and metal ions are governed by shared physicochemical principles. Generative models such as AlphaFold [36] and RosettaFold [37] generate structures of molecular complexes, but they do not explicitly learn representations of intermolecular interactions. A universal model for learning representations of molecular interactions would allow prediction of functional effects for uncharacterized molecules across molecular modalities.

A universal model for molecular interactions needs to capture the geometry of intermolecular interactions, imposed by hydrogen bonding, van der Waals forces, π-stacking, electrostatic interactions, and other physicochemical principles. However, each type of interaction varies in binding affinity, interface size, chemical composition, and functional roles. For example, protein-protein interfaces are often large, with median surface areas around 2,000–4,000 Å^2^, and are stabilized by multiple hydrophobic and electrostatic contacts. In contrast, protein-ligand binding sites are smaller, with median surface areas typically between 300 and 1,500 Å^2^, and are shaped by high-affinity, highly specific binding pockets [37, 38]. RNA and DNA interfaces introduce sequence-dependent constraints, and metal ions exhibit coordination-specific geometry.

We introduce ATOMICA, an all-atom geometric deep learning model that learns representations of intermolecular complexes across five molecular modalities: proteins, small molecules, metal ions, lipids, and nucleic acids, encompassing eight interaction types. ATOMICA is trained on a dataset of 2,037,972 interaction complexes (Fig. 1b), including 1,747,710 small-molecule interaction complexes from the Cambridge Structural Database (CSD) [39] and 290,262 molecular complexes from Q-BioLiP and the Protein Data Bank (PDB) [40–42]. The model uses a geometric, SE(3)-equivariant architecture and is trained with a self-supervised objective that combines denoising of atomic coordinates and masked block identity prediction. By learning from interactions that span proteins, nucleic acids, small molecules, and metal ions, ATOMICA generalizes across molecular modalities. We validate this cross-modality generalization by demonstrating improved representation quality and predictive performance in low-data modalities, such as protein-nucleic acid interactions, compared to models trained on a single pair of interacting modalities.

ATOMICA’s representations identify critical residues at interaction interfaces by measuring the change in latent embedding when individual blocks are masked, highlighting residues involved in intermolecular contacts. The latent space organizes complexes according to chemical similarity, grouping interfaces with similar physicochemical properties, such as hydrogen bonding or hydrophobic interactions. The model encodes compositional relationships, allowing algebraic operations in the latent space between binding partners, analogous to the semantic composition of natural language word embeddings. We use ATOMICA to construct interfaceome networks from predicted interactions among 5,856 protein-small molecule, 6,458 protein-ion, 6,649 protein-nucleic acid, 6,766 protein-lipid, and 17,158 protein-protein interfaces [43, 44]. These networks reveal 27 disease-associated pathways in ion, lipid, and small molecule networks, and identify candidate targets for autoimmune neuropathies. We apply ATOMICA to the dark proteome—regions of the proteome without functional annotation [45–47]. ATOMICA predicts 2,646 ligand-binding sites with putative ion or cofactor specificity, suggesting molecular functions in previously uncharacterized protein families. We experimentally confirm heme binding for five dark proteins using recombinant expression and spectroscopic assays.

## Results

### Dataset of molecular interactions for proteins, small molecules, ions, lipids, and nucleic acids.

We assembled a dataset of interacting molecular entities from the Cambridge Structural Database (CSD) v2022.3.0 [39] and Q-BioLiP [40, 41] which contains intermolecular interactions across all modalities available in the Protein Data Bank (PDB). From the CSD, we filtered for entries of small molecule crystals and extracted unique pairs of conformers from the unit cell (Methods 1), resulting in 1,767,710 interacting pairs of small molecules. We also processed 337,993 interaction complexes from Q-BioLiP, which includes structures of protein complexes with proteins, DNA, RNA, peptides, small molecules, and metal ions, as well as nucleic acid ligand structures from the PDB. The interaction interface between two entities is defined by atoms within an 8 Å distance to the other molecule to capture atoms in intermolecular bonds and the surrounding molecular context. For protein and nucleic acid containing complexes, we model only the amino acids and nucleic acids at the interface. In Fig. 1b, we show examples of the interaction interface for various complexes and their relative size.

We represent interaction complexes using two-level graphs that represent atomic-level details and higher-order chemical structures (Fig. 1c). At the first level, nodes in the graph represent atoms, each defined by its element type and 3D spatial coordinates. At the second level, atoms are grouped into chemically meaningful blocks, such as amino acids in proteins, nucleotides in nucleic acids, and functional moieties in small molecules, to form a block-level graph [48–50]. This hierarchical graph representation of intermolecular interactions captures local atomic interactions and broader structural organization and has theoretically higher expressive power than atom-level graphs alone [51]. Within each graph level, we define two types of edges: intramolecular edges connect the k nearest nodes in Euclidean space within each molecule, and intermolecular edges connect the k nearest nodes in Euclidean space on the interface of interacting molecules.

### ATOMICA: Geometric deep learning model of intermolecular interactions at atomic scale.

ATOMICA is a self-supervised geometric graph neural network that generates multi-scale embeddings at the atom, block, and graph level from the structure of interacting two molecules (Fig. 1c). Unlike modality-specific models, ATOMICA generates embeddings at the interface for any complex of interacting molecular modalities (small molecules, metal ions, amino acids, and nucleic acids) (Fig. 1b). ATOMICA can also be adapted by adding a task adaptor head and finetuning ATOMICA and the head for specific predictive tasks such as binding site annotation. Embeddings are learned through SE(3)-equivariant tensor field networks for message passing between nodes via the intermolecular and intramolecular edges (Methods 2). Tensor field networks have been used for learning interatomic potentials [52, 53], molecular docking [54], and scoring RNA structure [55].

To train ATOMICA, we use self-supervised pretraining objectives combining denoising and masked block prediction. Denoising serves as a powerful pretraining strategy for molecular property prediction [56–60]. In ATOMICA, denoising requires the model to reconstruct an interaction complex graph after it has been perturbed by a rigid SE(3) transformation and random torsion angle rotations of one molecular entity at the interface (Fig. 1e). This objective forces the model to learn the spatial relationships and physicochemical constraints that define intermolecular interactions, rather than memorizing specific atomic coordinates. ATOMICA is also trained to identify the type of randomly masked chemical blocks at the interaction interface. This masking objective, inspired by strategies for learning protein [2] and nucleic acid [61] sequence representations, encourages the model to infer missing chemical context based on the surrounding interface environment. These pretraining objectives drive ATOMICA to capture the geometric and chemical determinants of molecular recognition.

### ATOMICA captures multi-scale relationships and composition of interacting molecules.

**Figure 2:**
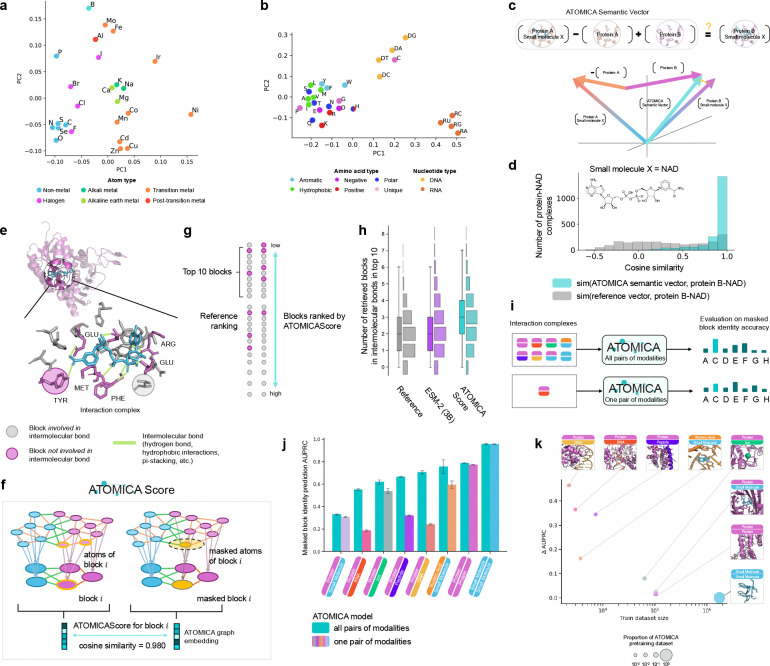
Analysing latent space of ATOMICA. **a** Principle components of mean embedding of elements in the pretraining validation and test set. **b** Principle components of mean embedding of amino acids and nucleotides in the pretraining validation and test set. **c** Compositional algebra of embeddings in the latent space of ATOMICA. **d** Similarity of ATOMICA Semantic Vectors to protein-NAD embeddings. Reference vectors are the embedding of a protein-small molecule complex chosen at random. **e** Some blocks in the interaction complex are involved in intermolecular bonds (hydrogen bonds, hydrophobic interactions, pi-stacking, etc.). **f** Definition of ATOMICAScore for block i, cosine similarity between interaction graph with block i masked and unmasked. **g** Nomination of blocks involved in intermolecular bonds at interfaces based on ATOMICAScore. The reference ranking is determined by random ordering of the blocks at the interaction interface. **h** Number of blocks involved in intermolecular bonds in the top 10 nominated blocks of ATOMICAScore, ESM-2 (3B parameters), and reference for protein-small molecule complexes in the pretraining test set. ATOMICA nominates more amino acids involved in intermolecular bonds than ESM-2 and the reference. **i** Schema to test generalizability of representations learned by ATOMICA trained on all pairs of modalities compared to models trained on one pair of modalities. We evaluate quality of representations based on masked block identity accuracy. **j** Masked block identity AUPRC for ATOMICA trained on all pairs of modalities and ATOMICA trained on one pair of modalities. ATOMICA trained on all pairs of modalities outperforms ATOMICA trained on one pair of modalities. Error bars represent standard deviation in AUPRC across 5 seeds for randomly masking blocks. **k** Increase in AUPRC between training on one pair of modalities and all pairs of modalities compared to dataset size of the one pair of modalities. Performance gains scale with dataset size increase, with largest improvements in AUPRC observed for pairs of modalities with the least data.

Visualizing ATOMICA embeddings at the graph level in 2D with UMAP for all interaction complexes shows that the embeddings capture relative chemical similarity of molecular interactions (Fig. 1f). The distribution of pairwise cosine similarities between protein-protein and protein-peptide graph embeddings is significantly higher than that between protein-peptide and all other interaction complexes (KS-statistic = 0.100, p-value < 0.001). The latent space of atom embeddings is organized according to periodic table location when projected into 2D with principal component analysis (PCA) (Fig. 2a). For PCA projections of block embeddings, the latent space organization resembles the chemical properties of the amino acids, which are also distinct from DNA and RNA nucleotides (Fig. 2b). Considering the pairwise cosine similarity of the graph embeddings between protein-small molecule interactions to the pairwise cosine similarity of the individual proteins and small molecules, the ATOMICA latent space of molecular interactions is complementary to embedding models that represent proteins (ESM-2 3B [2], Spearman correlation of −0.144, p-value < 0.001), and small molecules (Transformer-M [62], Spearman correlation of 0.107, p-value < 0.001) in isolation.

Molecular structures are compositional with protein domains determining functions [63], and the combination of these domains into multi-domain proteins [64]. We draw inspiration from the latent space properties of functional gene embeddings [65] and natural language embeddings in models such as word2vec, where algebraic relationships like $\vec{\text{King}}-\vec{\text{Man}}+\vec{\text{Woman}}$ yield an embedding close to $\vec{\text{Queen}}$ [66, 67]. To test if ATOMICA encodes similar compositionality, we consider a cofactor X that binds to both protein A and protein B. We evaluate whether the ATOMICA latent space supports the relationship: $ATOMICA\;Semantic\;Vector=\vec{\text{Protein}\;\text{A,}\;\text{Small}\;\text{Molecule}\;\text{X}}-\vec{\text{Protein}\;\text{A}}+\vec{\text{Protein}\;\text{B}}\approx \vec{\text{Protein}\;\text{B,}\;\text{Small}\;\text{Molecule}\;\text{X}}$ (Fig.2c). For unseen protein-NAD complexes with proteins sharing at most 30% sequence identity, the Semantic Vectors are significantly closer to the true embeddings of protein B-NAD complexes than to randomly chosen protein-small molecule complexes (KS statistic 0.599, p-value < 0.001, Fig.2d). Similar results are observed for other cofactors (Fig. S1).

### ATOMICA identifies residues on interaction interfaces involved in intermolecular bonds.

Zero-shot probing of ATOMICA shows it learns to differentiate between blocks involved in intermolecular bonds (hydrogen bonding, pi-stacking, hydrophobic interactions) against other blocks present at the interface (Fig. 2e). We define ATOMICAScore for block i as the cosine similarity between the ATOMICA embedding of the graph of the interaction complex with block i masked out and without masking (Fig. 2f). The blocks where masking causes the largest change in the representation have the lowest ATOMICAScore (Methods 6). ATOMICA is compared to a protein language model, which can predict the effects of mutations on protein fitness in a zero-shot manner [68], to assess whether it can identify amino acids essential for small molecule binding. For an unseen test set of protein-small molecule complexes, we evaluate precision at rank 10 and find that ATOMICAScore achieves the highest performance, with an average of 2.7 amino acid blocks in intermolecular bonds retrieved, followed by ESM-2 (3B) with 2.4, and the random reference with 2.0. Additionally, ATOMICA is able to retrieve amino acids across hydrogen-bonding, hydrophobic, and π-stacking intermolecular bond types (Fig. S2).

### Scaling laws drive generalizability of ATOMICA across molecular modalities.

To quantify the benefit of pretraining ATOMICA on multiple molecular modalities, we compare its performance to otherwise identical models pretrained exclusively on single pairs of interacting modalities (Fig. 2h). We evaluate each model using masked block identity prediction on a test set of interface complexes selected to have low sequence similarity and low chemical similarity to any training or validation examples (Methods 6). Pretraining ATOMICA across all modalities improves prediction accuracy for masked blocks in protein-DNA interfaces from 0.24 to 0.71 AUPRC (a 190% increase, with 2,253 protein-DNA training complexes). Similar improvements are observed for protein-RNA interfaces, where performance increases from 0.19 to 0.55 AUPRC using 2,975 protein-RNA training complexes. For protein-peptide interfaces, performance rises from 0.32 to 0.67 AUPRC with 7,346 training complexes. The improvements in AUPRC scale with dataset size (Fig. 2k). These results illustrate a scaling law: as the diversity and volume of interaction data increase, representation quality and predictive performance systematically improve. This scaling effect is analogous to observations in large language models, where expanding training data leads to predictable gains in generalization [69, 70]. The improvement in AUPRC is also consistent across the full range of residue burial (Fig. S3), showing that training ATOMICA on multiple molecular modalities improves recovery of masked blocks across interaction types. Stratifying performance by intermolecular context, defined by the average distance to nearest intermolecular blocks, pretraining across all pairs of interacting modalities yields a larger increase in performance for the blocks with less intermolecular context. For the blocks closest to other intermolecular blocks (8.0–8.4 Å away from 8 closest intermolecular blocks), there is a 13% improvement in AUPRC compared to a 28% improvement in AUPRC for blocks more distant to other intermolecular blocks (10.8–11.2 Å away from 8 closest intermolecular blocks) (Fig. S3b).

### Building interfaceome networks for five molecular modalities.

Disruptions in networks of interacting molecules, rather than defects in a single protein, often underlie disease [71]. Such disruptions can involve abnormal interactions among proteins, ions [72], small molecules [73, 74], lipids [75], or nucleic acids [76]. For disease d with associated proteins $V_{d}$, the disease pathway $H_{d}=\left(V_{d},E_{d}\right)$ is a subnetwork of the network of interacting proteins, where network relations are defined via protein-protein interactions and maps of cellular pathways [77, 78], albeit these networks do not directly capture interactions of proteins with other molecular modalities in the cell. We study disease pathways from a new perspective with ATOMICA by analyzing protein interactions with ions, small molecules, nucleic acids, lipids, and other proteins and their involvement in the same disease pathway.

**Figure 3:**
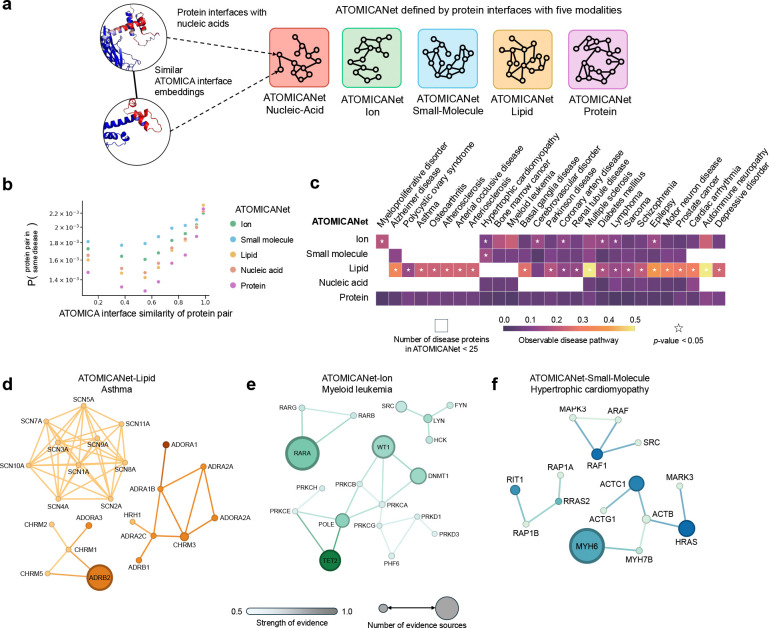
Interfaceome disease pathways on ATOMICANets. **a** Set up of the modality specific networks based on ATOMICA embedding similarity of protein interfaces with ions, small molecules, lipids, nucleic acids, and proteins. **b** Cosine similarity of ATOMICA embeddings of protein interface pairs across five interacting modalities compared to the probability of the protein pair being involved in the same disease. **c** Relative size of largest pathway component across diseases for each modality network. We display only the diseases which have statistically larger pathway components than expected in at least one ATOMICANet modality. The three largest pathway components for: **d** asthma in ATOMICANet-Lipid, **e** myeloid leukemia in ATOMICANet-Ion, **f** hypertrophic cardiomyopathy in ATOMICANet-Small-Molecule.

We probe the human interfaceome [18]–the set of human protein interfaces that mediate interactions with other molecules, including ions, small molecules, nucleic acids, lipids, and proteins–with ATOMICA to study disease pathways. ATOMICA-Interface is finetuned from ATOMICA for the embedding of protein interfaces (Methods 4). The putative interfaceome is obtained from predicted protein binding sites with ions, small molecules, nucleic acids, lipids, and proteins across 23,391 protein structures predicted by AlphaFold2 in the human proteome [18] (Methods 4). One ATOMICANet is constructed for each interacting modality, where nodes are proteins and edges connect proteins with high ATOMICA similarity between their modality-specific binding interfaces (Fig. 3a). The probability of pairs of nodes being involved in the same disease is higher if the two nodes have higher ATOMICA interface similarity (Fig. 3b), motivating exploration of the disease pathways present in ATOMICANets.

For the five modality-specific ATOMICANets, the number of proteins in the largest connected component (LCC) are as follows: ATOMICANet-Ion 5,831 proteins, ATOMICANet-Small-Molecule 5,246 proteins, ATOMICANet-Nucleic-Acid 5,974 proteins, ATOMICANet-Lipid 6,055 proteins, and ATOMICANet-Protein 15,450 proteins (network construction is described in Methods 4). The edges in the ATOMICANets are distinct from physical protein-protein interactions [79], with the average overlap in edges across the five ATOMICANets with PPI networks is 2.7%, with co-occurrence networks is 1.0%, with neighborhood networks is 0.3%, and across all sources of evidence (co-occurrence, database, experimental, fusion, neighborhood, and literature) is 6.4%.

Next we explore the disease pathways for 82 diseases by overlaying their disease-associated proteins onto the five ATOMICANets (full list of diseases available in Table S1). To analyze the observability of disease pathways in ATOMICANets, the size of the largest disease pathway component (proportion of disease-associated proteins in the largest connected component of the disease pathway [80]) is evaluated. For disease pathways–with at least 25 proteins in a ATOMICANet [71]–the average size of the largest pathway component for ATOMICANets are: Ion 11%, Small-Molecule 11%, Lipid 16%, Nucleic-Acid 10%, and Protein 6% (Fig. 3c).

### Examining disease pathways in interfaceome-based ATOMICANet networks.

Only diseases with disease-associated protein data coverage that exceeds a specific threshold have identifiable/observable disease pathways [71]. To assess the identifiability/significance of disease pathways in each ATOMICANet [71], we compare the size of the largest network component and the number of disconnected components in each disease pathway ($H_{d}$) to a non-parametric reference distribution given by randomized disease-associated proteins while preserving pathway size and node degree distribution (Methods 4).

First, for disease pathways in ATOMICANet-Lipid, of the 40 diseases with sufficient disease-associated proteins, 22 diseases exhibited significantly larger largest pathway components than expected, and 11 diseases had significantly fewer disconnected pathway components than expected. Asthma has 43 disease-associated proteins in ATOMICANet-Lipid and an observable disease pathway (Fig 3d, p-value < 0.001 for size of largest pathway component and p-value < 0.001 for number of pathway components; Benjamini-Hochberg with FDR control). The ATOMICANet-Lipid disease pathway involves proteins which share similar transmembrane domains, as proteins involved in the same disease tend to share subcellular localization [81–83]. Ten sodium channel family proteins (OpenTargets mean strength of evidence = 0.54, mean evidence sources = 5.2) are featured in the largest pathway component. In the second and third largest pathway components, there are 8 and 5 proteins, respectively, both involving G protein-coupled receptors (adenosine, α/β-adrenergic, muscarinic, and histamine receptors). These clusters have a mean strength of evidence of 0.61 and 0.56 with on average 66 and 245 sources of evidence [84], respectively. Proteins in these two components form key interactions with PIP2, a minority lipid component of the cell membrane [85].

Next, in ATOMICANet-Ion, 10 diseases had significantly larger pathway components than expected and 11 diseases had fewer disconnected pathway components than expected of the 35 diseases with sufficient disease-associated proteins. Myeloid leukemia has 53 disease-associated proteins in ATOMICANet-Ion (Fig 3e), of which 12 proteins are in the largest pathway component (p-value < 0.001), with a mean strength of evidence of 0.60 and on average 401 sources of evidence per disease protein [84]. This component includes TET2, a Fe^2+^ binder, which plays a key role in DNA demethylation. Four DNA binding proteins (DNMT1, POLE, WT1, PHF6) with similar ATOMICA embeddings possess zinc finger domains. Additionally, ATOMICANet-Ion connectivity in this disease pathway is observed between isoforms of protein kinase C and serine/threonine-protein kinase D which have a structurally conserved catalytic core that coordinates Mg^2+^-ATP.

In ATOMICANet-Small-Molecule, one disease has a significantly larger pathway component than expected of the 37 diseases with sufficient disease-associated proteins. Hypertrophic cardiomyopathy has 45 associated proteins in ATOMICANet-Small-Molecule (Fig 3f), and the largest pathway component is of size 7 (p-value = 0.037) with a mean strength of evidence of 0.70 and on average 630 sources of evidence per disease protein [84]. Proteins connected by ATOMICANet-Small-Molecule in this component share nucleotide (GTP/GDP, ATP/ADP) binding sites. These proteins include: myosin heavy chain proteins (MYH6, MYH7B) responsible for force generation in cardiac muscle [86, 87], cardiac actin (ACTC1) a crucial sarcomeric protein [88], and HRAS a GTPase regulating a host of signaling pathways and cellular responses [89].

For ATOMICANet-Nucleic-Acid and ATOMICANet-Protein networks, we do not observe any statistically significant pathway components. These networks also have relatively smaller largest pathway components with a mean size of 3.4 members for ATOMICANet-Nucleic-Acid and 6.0 for ATOMICANet-Protein, compared to 7.3 for ATOMICANet-Small-Molecule, 8.3 for ATOMICANet-Ion, and 8.9 for ATOMICANet-Lipid. Thus, disease pathways are likely currently unobservable in ATOMICANet-Nucleic-Acid and ATOMICANet-Protein [71].

### ATOMICANet associates lipid and ion interacting targets for autoimmune channelopathies.

In autoimmune peripheral neuropathy (APN) and multiple sclerosis (MS), the immune system attacks components of the nervous system. Ion signaling in neurons is essential for neural function [90–92], and disruptions in lipid metabolism are linked to disease onset [93]. To determine whether ATOMICANet-Lipid and ATOMICANet-Ion can identify disease-associated proteins, we use a diffusion-based network approach [94]. In this analysis, information is propagated from known disease-associated proteins across the network, and proteins are prioritized by how frequently they are reached during the diffusion process. Prediction performance is assessed by 5-fold cross-validation, using four folds for training and the fifth as held-out targets, repeated 100 times with different splits. A protein’s hit rate is the proportion of splits in which it appears among the top-ranked candidates identified by diffusion (Methods 4).

For multiple sclerosis, the diffusion-based approach applied to the five ATOMICANets identifies 69 of 119 disease-associated proteins. For autoimmune peripheral neuropathy, 40 of 44 disease-associated proteins are identified. KCNA1 and KCNA2 [95], voltage-gated potassium channels, were consistently ranked among the top candidates in 100% of random walks from all seed disease-associated proteins for MS and APN in ATOMICANet-Ion. Given that these disease-associated proteins are found in the cell membrane, ATOMICANet-Lipid also detects KCNA1 (70% hit rate for MS, 83% hit rate for APN) and KCNA2 (100% hit rate for MS, 100% hit rate for APS). The identification of these disease-associated proteins in ATOMICANets shows that ATOMICA embeddings of protein–ion and protein–lipid interactions capture disease-relevant associations for transmembrane ion channels in MS and APN. KCNQ3, a known component of the M current—a neuronal K^+^ conductance [91, 92]—was also highly associated with MS and APN in ATOMICANet-Lipid, appearing with a 100% hit rate. Several sodium channel alpha subunit family proteins [96] are also predicted to be associated with MS in ATOMICANet-Lipid, including SCN2A and SCN8A both with hit rates of 100%.

### Ligand annotation of ion and cofactor binding sites in the dark proteome.

**Figure 4:**
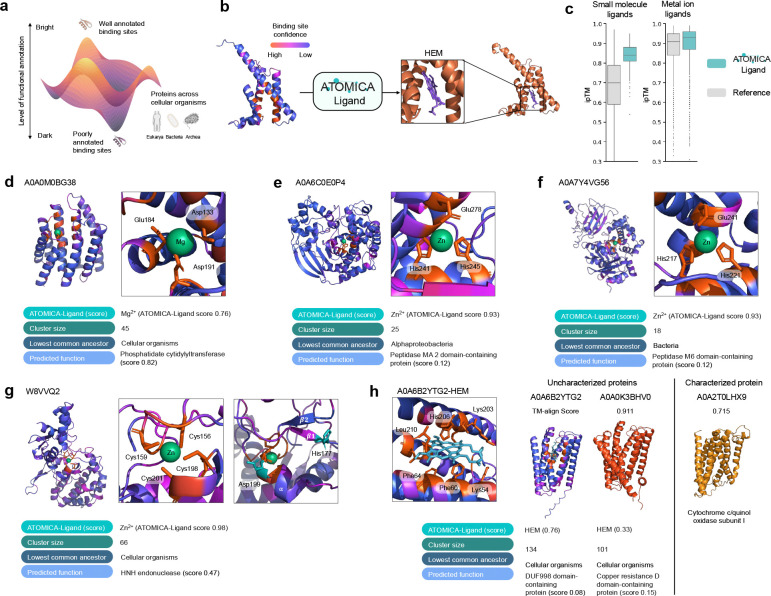
Dark proteome annotation with ATOMICA. **a** Annotation of binding sites for proteins across cellular organisms. **b** Prediction of ligands for metal ion and small molecule binding sites of proteins in the dark proteome. **c** AlphaFold3 ipTM scores of complexes from ATOMICA-Ligand annotated small molecule and metal ion compared to reference. Visualization of AlphaFold3-predicted structures for protein-ligand annotations made by ATOMICA-Ligand. Cluster size and lowest common ancestor are derived from Foldseek clustering of the AlphaFold Protein Structure Database [46]. Predicted functions are obtained from ProtNLM, a sequence-based protein function prediction model [105]. The following ATOMICA-Ligand annotations are shown: **d** Mg^2+^ annotation for A0A0M0BG38, a putative phosphatidate cytidylyltransferase, where Mg^2+^ binding is required for enzymatic activity. **e** A0A6C0E0P4 and **f** A0A7Y4VG56 are annotated with Zn^2+^ and predicted to be metallopeptidases. Both contain the conserved HEXXH Zn^2+^-binding motif characteristic of this family. **g** Zn^2+^ annotation for W8VVQ2, with the predicted binding site indicating a C4 zinc finger domain, a motif that remains undercharacterized in Bacteria. **h** Heme annotation for A0A6B2YTG2, with comparison to neighboring clusters A0A0K3BHV0 and A0A2T0LHX9 suggesting these uncharacterized clusters may contain putative cytochrome proteins.

Ion and cofactor binding sites are conserved functional features that are widely found throughout the proteome, despite significant differences in overall protein structure and sequence [97, 98]. With ATOMICA’s ability to represent molecular interactions, we extend its capabilities to annotate functional binding sites in regions of the proteome that currently lack any functional description, collectively known as the *dark proteome* [45–47]. Currently, 711,705 protein clusters (30.9% of all clusters cataloged in the AlphaFold (AFDB) FoldSeek) are *dark clusters* (Fig. 4a) [46, 99]. Proteins in these clusters have distinct structures and sequences, lack functional characterization, frequently include proteins with novel structural folds, and span all domains of life (Bacteria, Archaea, and Eukarya) [46, 100]. These dark clusters offer opportunities to discover new protein functions, reveal novel molecular mechanisms, and trace evolutionary paths underlying present-day protein diversity [45, 101, 102].

We finetune ATOMICA-Ligand from ATOMICA to annotate ligands to binding sites and apply it to proteins in dark clusters. Protein clusters are filtered for those with high-confidence AF2 structures (pLDDT > 90) resulting in 33,482 clusters. The representative AF2 member of each cluster is selected for further investigation. Ion and small molecule binding sites are identified on the surface of the protein with a deep learning-based classifier, PeSTo [18]. In total, 2,851 proteins are identified with ion binding sites and 969 proteins are identified with small molecule binding sites. ATOMICA-Ligand predicts ion and small molecule identities from protein pockets of PDB structures for 9 metal ions and 12 commonly found cofactors (Fig. 4b, Methods 5). Metal ions are annotated for 2,565 out of 2,851 proteins with ion binding sites and cofactors for 81 out of 969 proteins with small molecule binding sites. The quality of ATOMICA-Ligand predictions is confirmed with AlphaFold3 ipTM scores of the complexes. ipTM scores serve as a quantitative metric for the generation quality of complexes [103, 104]. The results from ATOMICA are statistically significantly higher than reference complexes for ions (KS Statistic: 0.11, p-value < 0.001) and small molecules (KS Statistic: 0.54, p-value < 0.001) (Fig. 4c). Reference complexes are determined by randomly assigning ions and cofactors to the predicted binding sites in the dark proteome. These protein annotations span 1,265 species, of which 1,051 are Bacteria, 99 are Eukaryota, and 115 are Archaea.

### Characterizing function through ion and cofactor binding sites in the dark proteome.

ATOMICA-Ligand predicts Mg^2+^ (ATOMICA-Ligand score 0.76) to an ion binding site in A0A0M0BG38 (Fig. 4d). This protein represents a cluster of 45 members that have the lowest common ancestor of cellular organisms. Sequence-based function prediction method, ProtNLM [105], predicts this is a phosphatidate cytidylyltransferase (score 0.82), an enzyme that combines cytidine triphosphate (CTP) and phosphatidate to produce CDP-diacylglycerol (CDP-DAG). These proteins are Mg^2+^-dependent enzymes for coordination with phosphorous groups, and it is in agreement with the ATOMICA-Ligand annotation of Mg^2+^ binding site.

For two uncharacterized Bacteria protein clusters, ATOMICA-Ligand annotates with high confidence Zn^2+^ (ATOMICA-Ligand score 0.93 for both proteins) to binding motifs which share similarity to metallopeptidases. A0A6C0E0P4 (Fig. 4e) is the representative protein for a cluster of size 25 with a lowest common ancestor of alphaproteobacteria. A0A7Y4VG56 (Fig. 4f) is the representative of a cluster of size 18 with a lowest common ancestor of Bacteria. The Zn^2+^-binding site in both proteins consists of two histidines and one glutamate, where the histidines are at positions i and i+4 on the same α-helix, while the glutamate is contributed by a separate α-helix. This Zn^2+^ binding HEXXH motif, which is present in most metallopeptidases, specifically in zincins it is consistent with the predicted Zn^2+^ -binding site [106, 107]. Structural alignment of the proteins with thermosylin (PDB: 2TLX) shows alignment of the Zn^2+^ binding motifs, and a TM-align score of 0.49 and 0.44 for A0A6C0E0P4 and A0A7Y4VG56, respectively.

ATOMICA-Ligand further characterizes a C4 zinc finger motif belonging to a protein in a cluster of previously uncharacterized bacterial proteins. Zinc fingers are frequently discovered in eukaryotes, but they are less characterized in Bacteria [108]. The ion binding site in W8VVQ2 is predicted to be a Zn^2+^ binding (ATOMICA-Ligand score 0.98). W8VVQ2 is part of a cluster with 66 members from Nonlabens marinus isolated from the Pacific Ocean [109]. For this cluster with ancient origins, there are 61 Bacteria C4 zinc fingers. The other 5 proteins in the cluster also contain zinc fingers with a less common 3Cys-1Glu motif. ProtNLM predictions suggest HNH endonuclease function (score 0.47), which features a ββα-metal topology [110]. This structure is identified in W8VVQ2 with the conserved histidine and aspartic acid of this motif [111] (Fig. 4g). These results highlight how ATOMICA has identified a putative novel class of bacterial C4 zinc fingers with endonuclease function.

Next, ATOMICA-Ligand identifies heme binding for ligand binding sites in ancient clusters of transmembrane proteins. We use ATOMICA-Ligand to annotate heme binding sites and characterize the TG2 and HV0 protein clusters of ancient origin to be putative transmembrane cytochrome proteins. A0A6B2YTG2 (abbr. TG2) is predicted to bind to heme with ATOMICA-Ligand score of 0.76, and is the representative of a cluster of size 134 with the cluster’s lowest common ancestor being cellular organisms (Fig. 4h). It is closely related to cluster A0A0K3BHV0 (abbr. HV0, TM-align score 0.911, ATOMICA-Ligand score of 0.33), comprised mostly of uncharacterized proteins and has 101 cluster members. We attempted to use the sequence function predictor, ProtNLM, to further elucidate protein function but it assigns domains of unknown function to TG2. For HV0, a low confidence prediction of a copper resistance D domain was made. However, ATOMICA predicts heme binding and the binding site predictor, PeSTo, does not find any high confidence metal ion binding sites for HV0. Further evidence to support heme-binding of these two clusters is that A0A7X0NTZ6–a member of the HV0 cluster–is a succinate dehydrogenase/fumarate reductase cytochrome B subunit protein where heme binding is necessary for electron transfer function [112]. Another related cluster with representative protein A0A2T0LHX9 (TM-align score with TG2 is 0.715) is annotated as cytochrome C/quinol oxidase subunit I additionally supports the heme-binding cytochrome annotation of TG2 and HV0 clusters.

### Experimental validation of heme binders predicted by ATOMICA.

Heme is a cofactor involved in electron transfer, oxygen transport, enzymatic catalysis, and cellular signaling. It features in 3.4% of protein-small molecule interaction complexes in the dataset. We use ATOMICA-Ligand to predict candidate heme binders in the dark proteome and select proteins for experimental validation. Nine proteins were selected and six were produced with sufficient quantity for testing of heme binding (Methods 7.1).

**Figure 5:**
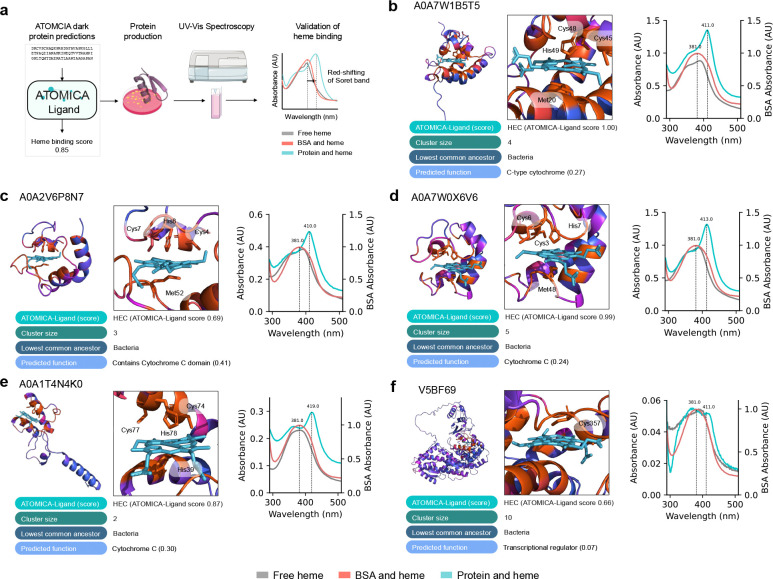
Experimental validation of dark protein heme binders predicted by ATOMICA-Ligand. **a** Selected proteins which are predicited by ATOMICA-Ligand to bind to heme are produced with recombinant protein expression (shown) and automated flow peptide synthesis. To confirm protein binding to heme, we use UV-Vis spectroscopy and the red-shifting of the Soret band at ~390 nm for free heme. The Soret band of heme and bovine serum albumin (BSA) is also shown as a negative control. The following dark proteins are confirmed to bind to heme **b** A0A7W1B5T5, **c** A0A2V6P8N7, **d** A0A7W0X6V6, **e** A0A1T4N4K0, and **f** V5BF69. The peaks of the Soret bands of BSA with heme, and protein with heme are labeled.

We use red-shifts in the Soret band from 390 nm for free heme to confirm the protein binding to heme (Fig. 5a). For five out of six proteins predicted by ATOMICA-Ligand to be heme-binding, we show experimental evidence of heme binding (Methods 7.2). Four of these experimentally confirmed proteins are predicted by ATOMICA-Ligand to exhibit covalent heme binding: A0A7W1B5T5 (ATOMICA-Ligand score 0.997), A0A2V6P8N7 (0.690), A0A7W0X6V6 (0.992), and A0A1T4N4K0 (0.874) (Fig. 5b–e). These predictions are in agreement with the presence of the heme binding CXXCH motif commonly found in cytochrome C proteins [113]. However, the presence of the CXXCH binding motif alone is insufficient to accurately predict heme binding as 19.2% of proteins in the dataset harboring this motif are not heme-bound.

For dark protein V5BF69 (0.663), which does not contain the canonical CXXCH heme binding motif, we show that it does bind heme experimentally (Fig. 5f). The sequence around the axial cysteine (C) coordinated to heme, AHGVCAG, is not captured by other common heme sequence motifs (GX[HR]XC[PLAV]G [113], FXXGXRXCXG [114]). While there is some overlap between the CAG subsequence in V5BF69 and the CXG subsequence found in common heme-binding motifs, only 4.1% of proteins in the dataset that contain the CXG subsequence are heme binders. This indicates that ATOMICA-Ligand can identify heme-binding motifs in the dark proteome that cannot be predicted by sequence motifs alone.

## Discussion

ATOMICA is a geometric deep learning model that represents intermolecular interactions by learning from over two million complexes involving small molecules, metal ions, amino acids, and nucleic acids. ATOMICA generates chemically informed embeddings that generalize across interaction types and exhibit compositionality in the latent space. These representations identify critical interface residues, recover interaction profiles linked to specific diseases, and enable systematic comparison across molecular classes. By constructing modality-specific interfaceome networks, ATOMICANets, we reveal disease modules across 27 diseases and identify disease-associated proteins, including those in autoimmune neuropathies, by integrating information from diverse interaction types. ATOMICA extends to the dark proteome, predicting and functionally annotating ion and cofactor binding sites in previously uncharacterized protein families. By modeling molecular interactions directly, ATOMICA provides functional insights based on similarities in interaction interfaces, rather than sequence or fold, and accurately predicts heme binding for five dark proteome proteins, which we validate experimentally.

A limitation of ATOMICA is its dependence on high-resolution molecular structures. Experimental structures are not available for all molecular complexes due to challenges such as crystallization, conformational flexibility, and molecular disorder. Advances in structure prediction, including AlphaFold3 [36] and RoseTTAFold [37], can generate high-confidence models for many molecular modalities, expanding the applicability of ATOMICA to regions without experimental coverage. However, some structural classes remain underrepresented. Non-globular domains, especially intrinsically disordered regions (IDRs), are poorly captured in current structural databases due to their flexibility [115]. Many IDRs undergo disorder-to-order transitions upon binding and are essential for cellular regulation and signaling [116]. Flexible regions such as antibody complementarity-determining regions are also critical for antigen recognition but are challenging to model accurately [117]. In these cases, relying only on structural input limits modeling accuracy and coverage. Integrating sequence-based features, which are informative for IDRs [118], could improve ATOMICA’s representations in structurally ambiguous regions.

Another limitation is the relatively small size of structural datasets compared to proximity-based interaction screening datasets. Techniques such as yeast two-hybrid [119], affinity purification mass spectrometry [120], DNA-encoded libraries [121], proximity ligation assays [122], fluorescence resonance energy transfer [123], and surface plasmon resonance [124] produce large-scale interaction data without 3D structures. These methods often capture transient interactions that are difficult to detect structurally. Incorporating these data types would extend the applicability of ATOMICA. Achieving this integration would require generating plausible structural models for interactions or developing multi-modal architectures that jointly learn from sequence, structure, and interaction data [125, 126].

ATOMICA models molecular interfaces, which creates opportunities for new applications of AI in biology. For example, ATOMICA embeddings can be used to predict the effects of sequence variation on binding interfaces and interpret variants in clinical and population-scale studies. The compositional latent space enables comparison and retrieval of interfaces, supporting the repurposing of ligands, cofactors, and binding domains. ATOMICA can also serve as a foundation for generative models that design interacting molecules, such as inhibitors, peptides, or nucleic acid aptamers, based on interface geometry. By embedding protein interfaces independent of specific ligands, ATOMICA can facilitate multitarget optimization and selectivity profiling in drug design.

Understanding molecular interactions is critical for deciphering cellular organization and disease mechanisms. To close the gap of limited coverage and isolated molecular representations, ATOMICA learns unified representations of interaction interfaces across molecular modalities. As the field advances toward building a virtual cell [127], models like ATOMICA will be valuable for capturing the spectrum of molecular interactions at atomic resolution.

## Methods

The Methods describe (1) the curation of datasets, (2) hierarchical all-atom graph neural network architecture of ATOMICA, (3) training ATOMICA, (4) studying disease pathways with ATOMICA, (5) the fine-tuning of ATOMICA for ion and ligand pockets, (6) metrics and statistical analyses, and (7) details on experimental validation of ATOMICA-predicted heme binders.

### 1 Curation of datasets

#### Small molecule structures.

We extract structures of small molecule interactions from the Cambridge Structural Database (CSD) v2023.2.0. The database was filtered for all CSD entries that satisfied the following criteria: organic, not polymeric, has 3D coordinates, no disorder, no errors, no metals, had only one SMILES string describing the crystal entry (in other words, each crystal is comprised of only one chemical compound), and molecules with 6–50 heavy atoms. CSD entries are unit cells of infinitely repeating crystal lattices. For our purposes of learning intermolecular interactions, we sampled many pairs of intermolecular interactions to represent all examples of intermolecular interactions in a given unit cell. Given an entry of the CSD, we iterate through each unique conformer in the unit cell and extract all pairs of interactions with neighboring peripheral conformers that are within 4 Å to the central conformer using the CSD Python API. In total, there are 1,767,710 structures of molecular pairs of 375,941 CSD entries. Inspired by fingerprint-based similarity measures used in chemistry [128], we use a one-hot encoding of the molecular complex from a vocabulary of 290 common chemical motifs [50] and Manhattan distance between the embeddings to sample 1,000 molecular complexes and their 100 nearest neighbors, giving a total of 10,000 molecular complexes for validation and test splits respectively, that are distinct from the training set.

#### General biomolecular structures.

We extract the structures of the interacting molecules from QBioLiP (June 2024), including structures of proteins that interact with ions, ligands, DNA, RNA, peptides, and proteins, and nucleic acids interacting with ions and ligands from the Protein Data Bank (PDB). For proteins, DNA, and RNA, we crop the complex to keep all residues within 8 Å to any atom, amino acid, or nucleic acid residue in the other molecule. In total, there are 124,541 protein-protein interaction complexes, 119,017 protein-small molecule interaction complexes, 74,514 protein-ion interaction complexes, 8,475 protein-peptide interaction complexes, 5,185 nucleic acid-ligand interaction complexes, 3,511 protein-RNA interaction complexes, and 2,750 protein-DNA interaction complexes. For protein-ion, protein-small molecule, protein-peptide, and protein-protein molecular complexes, we cluster each modality with 30% protein sequence similarity using MMseqs2 with a coverage of 80%, sensitivity of 8, and cluster mode 1 [129]. For protein-protein complexes, we also ensure that for any two complexes in different clusters, there is a maximum of 30% sequence similarity between all chains in the two complexes. For protein-RNA and protein-DNA complexes, we cluster by 30% protein sequence similarity and 30% nucleotide similarity using MMseqs2 with the same settings as above, this ensures that complexes in different clusters have a maximum of 30% protein sequence similarity and 30% nucleotide sequence similarity. For nucleic acid-ligand structures, we cluster based on 30% nucleotide sequence similarity. Finally, we split clusters into train, validation, and test splits using an 8:1:1 ratio.

#### Construction of hierarchical graphs of interacting molecules.

Given the atomic structure of two molecules interacting, an atom-level graph is constructed. Each atom in the complex maps to an atom node in the graph with the following features: element and 3D coordinates of the atom. Intramolecular atom edges are defined for each atom to the k nearest atoms in the same molecule. Intermolecular atom edges are defined for each atom to the k nearest atoms in the other molecule. In total, there are 118 atom types based on the elements of the periodic table.

Atom nodes are connected to block nodes. The block nodes have the following features: block type and 3D coordinates of the block given by the mean of the atomic coordinates of the atoms in the block. Each atom is connected to one block node. For proteins, peptides, DNA, and RNA, we define the atoms that belong to a given block by the amino acid and nucleotide residues. For small molecule ligands, blocks are defined by a vocabulary of 290 common chemical motifs. Atoms of sections of the molecule that cannot be fragmented into these motifs become blocks comprised of one atom. We follow the definition of Kong et al. [50] to compile a vocabulary and the fragmentation of molecules to blocks. Intramolecular block edges are defined for each atom to the k nearest blocks in the same molecule. Intermolecular block edges are defined for each atom to the k nearest blocks in the other molecule. In total, there are the following block types: 20 for canonical amino acids, 4 for DNA nucleotides, 4 for RNA nucleotides, 290 for small molecule fragments, and 118 for elemental blocks.

In addition, there are three special block types: mask, unknown, and global. The mask node is applied at pretraining for masked identity prediction of blocks. Unknown nodes are used for nodes that do not fall into the defined vocabulary, such as non-canonical amino acids and nucleotides. There are also two atom global-type nodes at the atom and block level. The two global nodes are connected to all nodes in each molecule at their respective level.

### 2 Hierarchical all-atom geometric deep learning model

#### Overview.

ATOMICA uses a SE(3)-equivariant 3D message passing network on graphs of molecular complexes to learn representations that are informative of the intermolecular interactions between molecules.

Given is a pretraining dataset of graphs of molecular complexes, $𝒟=\left\{G^{i}|i=1,\ldots ,N\right\}$. Our goal is to pretrain a model ℱ on 𝒟 such that it generates representations $h_{i}=ℱ\left(G^{i}\right)$ for every intermolecular complex $G^{i}$ that are chemically informative. In addition to representation learning, ℱ can also be finetuned on datasets of labeled graphs of molecular complexes, such as $𝒮=\left\{\left(G_{\text{target}}^{i},y_{i}\right)|i=1,\ldots ,M\right\}$, where M≪N to predict $y_{i}$ for every $G_{\text{target}}^{i}$.

#### Atom-level representation learning.

Here we outline the SE(3)-equivariant 3D message passing network for ATOMICA on the nodes of the graph $G^{i}$. Several rotational equivariant neural networks have been introduced for modeling molecules [52, 130–132]. We build on the E(3)-equivariant neural network layers presented by tensor-field networks implemented in e3nn [133] and DiffDock [54]. Message passing for the intermolecular edges and intramolecular edges is done separately, but the message passing framework for the two edge types is the same.

The feature vector of atom ($h_{a}^{\text{atom}}$) node a in $G^{i}$ is a geometric object comprised of a direct sum of irreducible representations of the O(3) symmetry group. The feature vectors $h_{a,(\lambda ,p)}^{\text{atom}}$ are indexed with λ,p, where λ=0,1,2,… is a non-negative integer denoting the rotation order and p∈{o,e} indicates odd or even parity, which together index the irreducible representations (irreps) of O(3). In ATOMICA model, we set $\lambda_{max}=1$ for $h_{a}^{\text{atom}}$, and we denote the number of scalar (0e) and pseudoscalar (0o) irrep features in $h_{a}^{\text{atom}}$ with ns, and the number of vector (1o) and pseudovector (1e) irrep features in $h_{a}^{\text{atom}}$ with nv.

The atom-type of node a, determined by the element of the atom, is embedded with a normal distribution and trainable weights as a scalar ns×0e. There are $L_{\text{GNN}}$ layers of message passing between atom nodes. At each layer l, the node updates for node a in the graph of interaction complex $G^{i}$ are given by:

(1) $$h_{a}^{\text{atom}}\leftarrow h_{a}^{\text{atom}}+LN\left(\frac{1}{\left|{𝒩}_{a}\right|}\sum_{b\in {𝒩}_{a}}Y\left({\hat{r}}_{ab}\right){⊗}_{\psi_{ab}}h_{b}^{\text{atom}}\right)$$

(2) $$\text{with}\;\psi_{ab}=\Psi \left(e_{ab},t_{ab},h_{a,(0e)}^{\text{atom}},h_{b,(0e)}^{\text{atom}}\right).$$

After each layer l of message passing, $h_{a}^{\text{atom}}$ is filtered down to irreps with $\lambda_{max}=2$. After L layers the $h_{a}^{\text{atom}}$ embedding is projected with a 2-layer MLP to a $d_{\text{node}}$-dimension vector.

#### Block-level representation learning.

The feature vector of block ($h_{b}^{\text{block}}$) node b in $G^{i}$ is also a geometric object defined in the same way as ($h_{a}^{\text{atom}}$). We initialize block nodes using a scalar, ns×0e, trainable embedding of block types.

Let $d_{\text{node}}$ be the dimension of $h_{b}^{\text{block}}$ and $n_{\text{heads}}$ be the number of attention heads. We define $d_{h}=d_{\text{node}}/n_{\text{heads}}$ as the dimension per head. The multi-head cross-attention operation can be expressed as:

(3) $$h_{b}^{\text{block}}\leftarrow h_{b}^{\text{block}}+MultiHead(h_{b}^{\text{block}},{\left\{h_{a}^{\text{atom}}\right\}}_{a\in A_{b}})$$

where $A_{b}$ is the set of atoms in block b, and MultiHead is defined as:

(4) $$MultiHead(h_{b}^{\text{block}},{\left\{h_{a}^{\text{atom}}\right\}}_{a\in A_{b}})=Concat\left({\text{head}}_{1},\ldots ,{\text{head}}_{n_{\text{heads}}}\right)W_{O}$$

and each head is computed as:

(5) $${head}_{i}=\sum_{a\in A_{b}}\alpha_{ba}v_{a}^{\text{atom,}(i)}\;\text{with}\;\alpha_{ba}=\frac{exp\;\left(q_{b}^{\text{block,}(i)}\;\cdot \;k_{a}^{\text{atom,}(i)}/\sqrt{d_{h}}\right)}{\sum_{v\in A_{b}}exp\;\left(q_{b}^{\text{block,}(i)}\;\cdot \;k_{v}^{\text{atom,}(i)}/\sqrt{d_{h}}\right)}$$

where $q_{b}^{\text{block,}(i)}=h_{b}^{\text{block}}W_{Q}^{\left(i\right)},\;k_{a}^{\text{atom,}(i)}=h_{a}^{\text{atom}}W_{K}^{\left(i\right)},\;v_{a}^{\text{atom,}(i)}=h_{a}^{\text{atom}}W_{V}^{\left(i\right)}$, and $W_{Q}^{\left(i\right)},\;W_{K}^{\left(i\right)},\;W_{V}^{\left(i\right)}\in R^{d_{\text{node}}\times d_{h}}$ and $W_{O}\in R^{d_{\text{node}}\times d_{\text{node}}}$. Message passing between the block nodes is specified by the same architecture as the atom nodes described in equation (1) and has separate model parameters.

#### Graph-level representation learning.

To pool $h_{b}^{\text{block}}\in R^{d}$ for $b\in G^{i}$ for a graph-level representation $h_{i}^{\text{graph}}\in R^{d}$, we use multi-head self-attention for $L_{\text{pool}}$ layers and sum the output $h_{b}^{\text{block}}$ for all $b\in G^{i}$ for $h_{i}^{\text{graph}}$.

#### Self-supervised learning with denoising and block identity masking.

Node-level denoising as an objective function has been useful for pretraining on 3D coordinate molecular datasets from DFT-generated molecules to prevent over-smoothing of GNNs [134], and it has proven that it is related to learning a force field of per-atom forces [8, 135]. In addition, denoising is linked to score-matching which has also been popular in training generative models [54, 136] as well as unsupervised binding affinity prediction [60]. Thus, this motivates the application of denoising as an objective for self-supervised training.

Given $G^{i}\in 𝒟$, which is comprised of atom and block nodes from two interacting molecules. ${\tilde{G}}^{i}$ is a perturbed graph created by applying two transformations to a molecule in $G^{i}$:

- Rigid rotation and translation: A rotation vector is sampled $\omega ∼p(\omega )={𝒩}_{SO(3)}$ and we apply the rotation of all atom and block coordinates about the center of the selected molecule. A translation vector is sampled $t∼p(t)=𝒩\left(0,\sigma_{t}^{2}I\right)$ and we apply this translation to all atom and block coordinates of the selected molecule.
- Torsion angle noising: Torsion angles are sampled $\theta ∼p(\theta )={𝒩}_{SO(2{)}^{m}}$ where m is the number of rotatable bonds in the molecule. For peptides, proteins, RNA and DNA we only perturb rotatable bonds in the side chain.

To predict the rotation score $s_{\omega }\in R^{3}$ and the translation score $s_{t}\in R^{3}$ from ${\tilde{G}}^{i}$, the node representations at the atom and block level are convolved with the center of the graph using a tensor field network [54]:

(6) $$s\leftarrow LN\left(\frac{1}{\left|{𝒜}^{\prime }\right|}\sum_{a\in {𝒜}^{\prime }}Y\left({\hat{r}}_{ca}\right){⊗}_{\phi_{ca}}h_{a}^{\text{atom}}\right)\;\text{with}\;\phi_{ca}=\Phi \left(e_{ca},h_{a,(0e)}^{\text{atom}}\right),$$

where node $a\in {𝒜}^{\prime }$ are the atom nodes in the perturbed molecule and c is the center of the perturbed molecule. This is a weighted tensor product, with the weights given by a 2-layer MLP, Φ, which takes as input the Gaussian smearing $d_{\text{edge}}$-embedding of the Euclidean distance between coordinates of the center c and node a, and the scalar component of $h_{a}^{\text{atom}}$.

Finally, the rotation score is given by the pseudovector irrep component $s_{\omega }=\Gamma_{\omega }(h_{i}^{\text{graph}})\;*\;S_{\left(1e\right)}$ and the translation score is given by the vector irrep component $s_{t}=\Gamma_{t}(h_{i}^{\text{graph}})\;*\;s_{\left(1o\right)}$, where $\Gamma_{\omega }$ and $\Gamma_{t}$ are 2-layer MLPs that project the graph representation of $G^{i}$ to a single scalar.

To predict the torsion score $s_{\theta }\in R^{m}$, the atom nodes are convolved with the center of the rotatable bonds connecting atoms $a_{z0},\;a_{z1}$. Let z denote the center of one of the rotatable bonds. We connect ${𝒩}_{z}=\{a|a\in {𝒜}^{\prime },\left\|{\hat{r}}_{za}\right\|<5Å\}$ which are all atoms in the perturbed molecule within 5 Å to the center of the bond:

(7) $$h_{z}=\frac{1}{\left|{𝒩}_{z}\right|}\sum_{a\in {𝒩}_{z}}\left(Y^{2}\left({\hat{r}}_{z}\right)⊗Y\left({\hat{r}}_{za}\right)\right){⊗}_{\pi_{za}}h_{a}^{\text{atom}}\;\text{with}\;\pi_{za}=\Pi^{\left(t\right)}\left(e_{za},h_{a,(0e)}^{\text{atom}},h_{a_{z0},(0e)}^{\text{atom}}+h_{a_{z1},(0e)}^{\text{atom}}\right),$$

The first tensor product is between the second order irreps of the unit direction vector along the two atoms $a_{z0},\;a_{z1}$ of the bond $z,\;Y^{2}\left({\hat{r}}_{z}\right)$, and the unit direction vector between the center of the bond and atom $a,\;Y\left({\hat{r}}_{za}\right)$. This is followed by a weighted tensor product with the weights given by a 2-layer MLP, Π, which takes as input the Gaussian smearing $d_{\text{edge}}$-embedding of the Euclidean distance between coordinates of the bond center z and node a, the scalar component of $h_{a}^{\text{atom}}$, and the sum of the scalar components of the two atoms in the bond, $h_{a_{z0}}$ and $h_{a_{z1}}$. Finally, we sum the scalar and pseudoscalar components of $h_{z}$ and project it to a single scalar $s_{\theta_{z}}$ using a 2-layer MLP.

We calculate the loss components as follows:

(8) $$l_{\omega }={\left\|s_{\omega }-\nabla_{\omega }\;log\;p(\omega )\right\|}^{2}$$

(9) $$l_{t}={\left\|s_{t}-\nabla_{t}\;log\;p(t)\right\|}^{2}$$

(10) $$l_{\theta }=\sum_{z}{\left\|s_{\theta_{z}}-\nabla_{\theta_{z}}log\;p\left(\theta_{z}\right)\right\|}^{2}$$

where $\nabla_{t}log\;p(t)=-t/\sigma_{t}^{2}$. The values of $\nabla_{t}log\;p(t)$, $\nabla_{\theta_{z}}log\;p\left(\theta_{z}\right)$ can be calculated by pre-computing a truncated infinite series following [54, 60].

In addition to denoising, we also pretrain the model by masking out block identities and predicting the masked block identities. For each graph $G^{i}$, 10% of blocks are randomly sampled and their block identities are replaced with the special ‘mask’ block and we denote these blocks as ℬ. For a masked block b∈ℬ, the probability vector of the block identity is predicted with ${\overset{ˆ}{y}}_{b}=Softmax\left(\Upsilon \left(h_{b}^{\text{block}}\right)\right)$, where Υ is a 2-layer MLP. We calculate the masked loss using a cross-entropy loss:

(11) $$l_{m}=-\frac{1}{\left|ℬ\right|}\sum_{b\in ℬ}y_{b}\cdot log\left({\overset{ˆ}{y}}_{b}\right)$$

The pretraining loss is then calculated by a weighted sum of the above loss functions:

$$ℒ=\beta_{\omega }l_{\omega }+\beta_{t}l_{t}+\beta_{\theta }l_{\theta }+\beta_{m}l_{m}$$

### 3 Training details for ATOMICA

#### Overview.

We pretrain ATOMICA on the training split of biomolecular structures and small molecule structures to generate embeddings of molecular complexes at the atom, block, and graph scales. To learn representations in a self-supervised manner, during training, we apply noise to the atomic coordinates and mask block identities of the input graphs of the molecular complex. At inference time, embeddings from the graphs are generated without noise or masked blocks.

#### Hyperparameter tuning.

We employed a hyperparameter optimization strategy utilizing Ray Tune [137] in conjunction with Optuna [138] and the Asynchronous Successive Halving Algorithm (ASHA) scheduler [139]. The hyperparameter space we search on includes: the number of nearest neighbors to define edges to in the graph k∈[4,8,16], dropout in the tensor field network ∈[0.00,0.01,0.05,0.10], edge dimension $d_{\text{edge}}\in [16,24,\;32]$, node dimension $d_{\text{node}}\in [16,\;24,32]$, and the number of tensor field network layers L∈[4,6,8]. The best hyperparameters are shown in bold and chosen based on the lowest validation loss when trained on a random 10% subsample of the training set. Then for determining the level of noise to apply to the interaction complexes, we conducted a second hyperparameter search on rotation $\sigma_{\omega }\in [0.25,0.5,\;1]$, $\omega_{max}\in [0.25,0.5,1]$, translation $\sigma_{t}\in [0.5,1,1.5]$, and torsion $\sigma_{\theta }\in [0.25,0.5,1]$. The best hyperparameters are shown in bold and chosen based on the highest masked block identity prediction accuracy when trained on a random 10% subsample of the training set. For the loss function, we set $\beta_{\omega }=1,\;\beta_{t}=1,\;\beta_{m}=0.1$, and the block identities are randomly masked at 10% probability. ATOMICA is trained on the full training set with the above hyperparameters, the learning rate cycles between 1e-4 and 1e-6 using Cosine Annealing Warm Restarts, with a cycle length of 400,000 steps, and the model is trained for 150 epochs.

#### Implementation.

ATOMICA is implemented with PyTorch (Version 2.1.1) [140] and PyTorch Geometric (Version 2.1.1) [141]. Training runs were monitored with Weights and Biases [142]. Models are trained on 4 NVIDIA H100 Tensor Core GPUs in parallel.

### 4 Analyses of disease pathways with ATOMICA

#### Overview.

Following pretraining ATOMICA, we finetune the learned representations of the molecular complexes for diverse biomedical downstream tasks. We demonstrate ATOMICA’s ability to fingerprint protein surfaces to a diverse set of binders: ions, ligands, proteins, nucleic acids, and lipids. We demonstrate the versatility of these embeddings by studying the presence of disease pathways in networks constructed from these embeddings, showing that across interacting modalities similar ATOMICA embeddings are likely to be involved in the same disease.

#### Training ATOMICA-Interface.

To support the embedding of protein binding interfaces, we fine-tune ATOMICA with structures of interfaces rather than complexes. For our finetuning dataset, we adapt the biomolecular structures from the training set. For each graph $G^{i}$ we crop the graph to only the protein interface of one protein in the complex $G^{i\prime }$. Let $h_{i}^{\text{graph}}=ℱ\left(G^{i}\right)$ where ℱ is pretrained and frozen ATOMICA. Our goal is to train 𝒢 initialized with ℱ such that $h_{i}^{\text{graph}\;\prime }=𝒢\left(G^{i\prime }\right)$ for every intermolecular complex $G^{i}$ with $h_{i}^{\text{graph}}$ and $h_{i}^{\text{graph}\;\prime }$ are aligned. Then for a randomly sampled mini-batch of $N_{\text{batch}}$ examples, the loss function is:

(12) $${ℒ}_{\text{interface}}=-\;\sum_{i=1}^{N_{\text{batch}}}log\frac{exp\;\left(sim\;\left(h_{i}^{\text{graph}},\;h_{i}^{\text{graph}\;\prime }\right)/\tau \right)}{\sum_{j=1}^{N_{\text{batch}}}1_{\left[j\neq i\right]}\;exp\;\left(sim\;\left(h_{i}^{\text{graph}},\;h_{j}^{\text{graph}\;\prime }\right)/\tau \right)}\;\;+log\frac{exp\;\left(sim\;\left(h_{i}^{\text{graph}\;\prime },\;h_{i}^{\text{graph}}\right)/\tau \right)}{\sum_{j=1}^{N_{\text{batch}}}1_{\left[j\neq i\right]}\;exp\left(sim\;\left(h_{j}^{\text{graph}},\;h_{i}^{\text{graph}\;\prime }\right)/\tau \right)}$$

where sim is cosine similarity and τ is the temperature factor. This contrastive loss is adapted from the normalized temperature-scaled cross-entropy loss [143]. We finetune the model for 50 epochs with a cyclic learning rate ranging from 1e-3 to 1e-5 over 50000 steps. Three replicates of the model are trained. The models were finetuned on 4 NVIDIA H100 Tensor Core GPUs in parallel.

#### Detection of binding sites across the human proteome with PeSTo.

We employ PeSTo (Version 4.1) [18], which for a given protein structure, PeSTo predicts the probability of each amino acid as a binding site for an ion, ligand, nucleic acid, protein, and lipid binder. PeSTo is run across all human proteins from the AlphaFold Protein Structure Database [99, 144]. For each protein and binding modality, we extract binding sites as all amino acids with PeSTo confidence > 0.7 and AlphaFold2 pLDDT > 70 with at least 5 amino acids at the binding site to keep only high-confidence binding sites. This gives us a total of 6,458 protein-ion binding interfaces, 5,856 protein-ligand binding interfaces, 6,649 protein-nucleic acid binding sites, 6,766 protein-lipid binding sites, and 17,158 protein-protein binding interfaces.

#### Construction of ATOMICANet.

All binding sites for each modality extracted with PeSTo are embedded with ATOMICA-Interface. We compute pairwise cosine similarity matrices from the embeddings for each of the three ATOMICA-Interface replicates and then average them to produce a single, consolidated cosine similarity matrix. Using the resultant cosine similarity matrix, we then construct a network for each modality based on a cosine similarity threshold and enforce that each node in the network has a maximum degree of 50. Cutoffs are defined such that 90% of the proteins in each modality are in the largest connected component. We construct these networks using NetworkX [145]. In total, for the largest connected component in each network, we have 5,831 nodes in ATOMICANet-Ion, 5,246 nodes in ATOMICANet-Small-Molecule, 5,974 nodes in ATOMICANet-Nucleic-Acid, 6,055 nodes in ATOMICANet-Lipid, and 15,450 nodes in ATOMICANet-Protein. Visualisations of the networks are constructed with Gephi [146].

#### Dataset of disease target proteins.

We extract targets for diseases from Open Targets (2024–09) [84]. Genes are associated with diseases using multiple lines of evidence (genetic association, somatic mutations, known drug, affected pathway) and we use the overall score, which is an aggregated sum of all evidence sources. For all diseases, we keep all targets with overall evidence scores > 0.5. The list of 82 diseases can be found in Supplementary Table 1.

#### Observation of disease pathways in ATOMICANets.

For the target-disease associations, we study their disease pathways across the five ATOMICANets. A disease pathway is one or more connected subgraphs comprised of disease proteins [71], with a minimum requirement of 25 associated genes for a disease for there to be an observable disease pathway. We refer to a disease d with associated proteins in a modality network $V_{d}^{\text{modality}}$ and the disease pathway is the undirected subgraph $H_{d}^{\text{modality}}=\left(V_{d}^{\text{modality}},E_{d}^{\text{modality}}\right)$. Following [80], we use their definition of the size of the largest pathway component as the fraction of disease proteins that lie in $H_{d}^{\text{modality}}$ ‘s largest connected component. For all modalities with $\left|V_{d}^{\text{modality}}\right|>25$, we analyze the size of the largest pathway component. To assess the statistical significance of the observed pathway size and number of disconnected components, we compared it against a distribution derived from the observed pathway size and number of disconnected components of 1,000 randomized sets of disease proteins. These randomized sets were constructed to match the degree distribution of the original disease proteins, thereby accounting for the heterogeneous connectivity patterns in ATOMICANets. For each network, we applied the Benjamini-Hochberg procedure to correct for multiple hypothesis testing, considering results with adjusted p-values < 0.05 as statistically significant.

#### Disease pathway detection in ATOMICANets.

Given ATOMICANets and the disease proteins, the task is to predict new proteins that are likely associated with the disease. This has been widely explored on PPI networks, with diffusion-based methods demonstrating strong performance [80, 147]. We use a variant of random walks with restarts: a random walker starts from a given seed protein, and at every time step, it can move to neighboring nodes or restart from a seed node. The probability of a walker being at node i at time t is then given by $p^{t}=(1-r)Ap^{t-1}+rp^{0}$, where $p^{0}$ is the initial probability, which is equal probability to start from each seed node, r=0.75 is the restart probability, A is the normalized adjacency matrix. We run the random walk until $\left\|p^{t}-p^{t-1}\right\|<10^{-6}$ and t<1000. The nominated proteins are then the 500 proteins with the highest probability of being visited. For each disease, modality, and random seed, we set up a 5-fold cross-validation split on $V_{d}^{\text{modality}}$; for each fold, we set them to be disease proteins to be detected, and the proteins in the remaining folds are set as seed nodes. This is repeated for 100 independent seeds, and the hit rate is calculated as the proportion of seeds for which the disease protein was detected.

### 5 Dark proteome binding site analysis with ATOMICA-Ligand

#### Overview.

We demonstrate versatility in ATOMICA and finetune the model for annotating ions and ligands to binding sites. The finetuned version of the model is applied to putative binding sites in the dark proteome.

#### Training ATOMICA-Ligand.

The objective is to predict the probability of a specific ion or ligand binding to a given protein interface pocket. We frame this as a binary prediction task and finetune a separate model for each ion and small molecule. A predictive head is a $L_{\text{ligand}}$-layer MLP. For each ion and small molecule, we use RayTune with Optuna and ASHA to finetune ATOMICA-Ligand from ATOMICA and find the optimal hyperparameters among $L_{\text{ligand}}\in [3,\;4,\;5]$, learning rate $\in \left[10^{-6},10^{-3}\right]$, non-linearity ∈[relu,gelu,elu], hidden dimension of MLP∈[16,32,64], gradient clipping ∈[None,1], and the number of nearest neighbors to define edges to in the graph k∈[4,6,8]. To address class imbalances in our dataset, we apply a weighted sampling strategy during training, where each protein pocket receives a sampling weight inversely proportional to the total count of its label class. For each ion and small molecule, we finetune ATOMICA-Ligand for 50 epochs on 1 NVIDIA H100 Tensor Core GPU. Three replicate models are trained for each ion and small molecule. For binary classification of binding sites, we set thresholds that maximize the F1 score, constraining these values to fall within the range of 0.05 to 0.95.

#### Dataset curation.

Given an ion or small molecule, we separate all graphs in the pretraining set containing this ion bound to a protein. We cluster protein binders with a 30% protein sequence similarity cutoff, coverage of 80%, sensitivity of 8, and cluster mode 1 using MMseqs2 [129]. The clusters are then divided into training, validation, and test sets in an 8:1:1 ratio. We set up this split for the following metal ions: Ca, Co, Cu, Fe, K, Mg, Mn, Na, Zn, and the following small molecules with these PDB chemical codes: ADP (adenosine diphosphate), ATP (adenosine triphosphate), CIT (citric acid), CLA (chlorophyll A), FAD (flavin adenine dinucleotide), GDP (guanosine diphosphate), GTP (guanosine triphosphate), HEC (heme C), HEM (heme B), NAD (nicotinamide adenine dinucleotide), NAP (NADP+, nicotinamide adenine dinucleotide phosphate, oxidized form), NDP (NADPH, nicotinamide adenine dinucleotide phosphate, reduced form).

#### Dark proteome annotation.

The dark proteome is comprised of proteins that are dissimilar in sequence and structure from all currently annotated proteins. We use the clusters of the dark proteome from FoldSeek cluster on the AlphaFold Protein Structure Database [46]. We limit our analysis to the 33,482 clusters with an average pLDDT > 90. For each cluster, we take the representative protein and run PeSTo on the protein structure to predict ion and small molecule binding sites. We keep residues with PeSTo confidence > 0.8 as the putative binding site, with a minimum of 5 residues required. In total, we extract 2,851 ion binding proteins and 969 small molecule binding proteins from the 33,482 representative proteins. Given these binding interfaces, we run ATOMICA-Ligand for all finetuned ion and small molecules to annotate chemical identities to the binding sites. We evaluated the quality of ATOMICA-Ligand predicted protein-ligand complexes by folding them with AlphaFold3 and evaluating their ipTM scores. For comparison, we established a reference baseline using randomly sampled proteins from the dark proteome with predicted ion and small molecule binding capabilities. These reference proteins were selected and paired with ligands to match both the number and identity of annotated ligands in our predicted complexes. For sequence-based annotation we run the Google Colab notebook with ProtNLM [105].

### 6 Additional quality metrics and statistical analyses

#### Visualization of embeddings.

We embed 2,105,459 molecular interaction complexes from training, validation, and testing datasets, and project these embeddings into two dimensions using Uniform Manifold Approximation and Projection for Dimension Reduction (UMAP) [148] with 30 neighbors and a minimum distance of 0.001. All interaction complexes were visualized with PyMOL [149].

#### Visualization of atom and block identity.

From molecular interaction complexes in training, validation, and testing datasets, we take the element-wise average atom and block embeddings for each identity. For the atom and block embeddings, we projected the embeddings into two dimensions using Principal Component Analysis (PCA).

#### Composition of ATOMICA embeddings.

We analyze the composition of protein-small molecule embeddings for interaction complexes in the test set. To get the embedding of the pocket only, we remove the atom and block nodes corresponding to the small molecule and embed the graph with ATOMICA. We evaluated our algebraic composition method by measuring the cosine similarity between ATOMICA Semantic Vectors and their corresponding original complexes. For reference, we compared this similarity against a reference distribution obtained by calculating the cosine similarity between the original complexes and embeddings of randomly sampled protein-small molecule complexes from the test set. This comparison framework enables us to evaluate whether our composition method generates embeddings that capture the properties of the actual complexes. This analysis was conducted for the following test sets of small molecule complexes: NAD (218 complexes), FAD (215 complexes), HEM (168 complexes), PLP (157 complexes), and ATP (105 complexes).

#### Capturing intermolecular bonds with ATOMICAScore.

To identify amino acids involved in intermolecular interactions, we analyzed protein-small molecule complexes in the test set using PLIP [150]. We then quantified the contribution of each amino acid to the interaction by calculating an importance score, ATOMICAScore. For amino acid i, ATOMICAScore is defined as $a_{i}=sim\left(h_{G},h_{G_{i}^{\text{mask}}}\right)$, where $h_{G}$ is the embedding of the original complex, and $h_{G_{i}^{\text{mask}}}$ represents the embedding of the modified complex in which amino acid i has been replaced with a special mask token and its constituent atoms substituted with a single special mask atom. Here, sim denotes the cosine similarity between the two embedding vectors. In total, we analyze 5,691 protein-small molecule complexes with at least 20 amino acid blocks at the interface. Within the 10 amino acids with the lowest ATOMICAScores we count the number of amino acids involved in intermolecular bonds. For reference, we compare this to randomly nominating 10 amino acids at the interface, and to ESM-2 (3 billion parameters) [2] and score amino acids by the log likelihood of the masked mutant compared to the original amino acid [68].

#### Training ATOMICA on a single pair of interacting modalities (modality-specific training).

To demonstrate representations learned by ATOMICA are generalizable across multiple modalities, we train models with identical architecture and hyperparameters on only single pairs of interacting modalities (small molecules, protein-ion, protein-small molecule, protein-DNA, protein-RNA, protein-peptide, protein-protein, nucleic acid-small molecule). Using the same training setup as ATOMICA, these models are trained on the same training data as ATOMICA but filtered for only one pair of interacting modalities. The models are trained for 150 epochs on 4 NVIDIA H100 Tensor Core GPUs in parallel. The model checkpoint with the lowest validation loss is then used for further finetuning on masked block identity prediction on the same training data for 50 epochs with a learning rate of 1e-4. We also finetune ATOMICA for 50 epochs on block identity prediction for each pair of interacting modalities. To compare the quality of embeddings generated by ATOMICA and versions of it trained on single modalities, we evaluate the accuracy of masked block identity prediction on a test set. This test set was not seen by any of the models and has 30% sequence similarity and minimal small molecule fingerprint similarity to any training and validation data.

## 7 Experimental validation of heme binders

### 7.1 Selection of heme binders for experimental validation

Of the 59 proteins predicted to bind to heme non-covalently (HEM ligand) and eight proteins predicted to bind to heme covalently (HEC ligand) from the dark proteome, nine were selected for experimental validation. In addition to their ATOMICA-Ligand confidence score, they were selected based on surpassing a minimum AlphaFold3 ipTM score of 0.85 and the absence of a large transmembrane domain, which would make protein synthesis difficult. The UniProt identifiers of these proteins are: A0A7W1B5T5, A0A1T4N4K0, A0A4P5TA35, A0A7W0X6V6, A0A2V6P8N7, A0A136KY61, A0A7Y8LED7, A0A7V7N0X5, and V5BF69.

### 7.2 Synthesis of proteins

A0A7W1B5T5 and A0A1T4N4K0 were synthesized with automated flow peptide synthesis. In addition, all nine proteins were synthesized and purified as recombinant proteins by GenScript (Piscataway, NJ, USA).

#### Reagents and supplies.

Disposable fritted polypropylene reaction vessels were purchased from Torviq (cat. no. SF-0500-LL for 6 mL reactors). H-RAM-ChemMatrix resin (0.17 mmol/g, batch no. 18007556) was purchased from ChemMatrix. Fmoc-amino acids were purchased from the Novabiochem product line of Millipore Sigma and were used as received. OmniSolv^®^ grade *N,N*-Dimethylformamide (DMF, biosynthesis grade) was purchased from Millipore Sigma (product DX1732–1) and was equipped using AldraAmine trapping agents (for 1000–4000 mL DMF, catalog number Z511706). *N,N*-Diisopropylethylamine (DIPEA; ReagentPlus ≥ 99%), piperidine (ACS reagent, ≥ 99.0%), trifluoroacetic acid (HPLC grade, ≥ 99.0%), triisopropylsilane (TIPS, ≥ 98.0%), acetonitrile (ACN, HPLC grade), 1,2-ethanedithiol (EDT, 98+%), trimethylsilyl chloride (TMS-Cl, 98.0% GC), triphenylphosphine (PPh3, ReagentPlus 99%) and formic acid (FA, ≥ 95.0%) were all purchased from Sigma-Aldrich and used as is. O-(7-azabenzotriazol-1-yl)-*N,N,N’,N’*-tetramethyluronium hexafluorophosphate (HATU, ≥ 97.0%) was purchased from P3 Biosystems. Dichloromethane (DCM, HPLC grade > 99.9%) was purchased from Fisher. Hemin (BioXtra, ≥ 96.0%, cat. no. 51280–1G) was purchased from Sigma-Aldrich. Water was deionized in-house using a Milli-Q water purification system from Millipore.

#### Automated flow peptide synthesis.

125 mg of H-RAM ChemMatrix^®^ resin was weighed in a 6 mL Luer Torviq reactor and swollen using DCM (15 min) and DMF (15 min). The reactor was drained and deposited in the heating reactor of the “Amidator”; an automated-flow peptide synthesiser built in the Pentelute Lab. Sequences for A0A7W1B5T5 and A0A1T4N4K0 were directly taken from UniProt. Synthesis was carried out as previously described [151, 152]. After the synthesis, the resin was drained, washed with DCM ×3 and let to dry on the vacuum manifold.

#### Cleavage protocol.

Half of the peptide-anchored resin was transferred to a 10 mL Luer-Lock Torviq syringe equipped with a frit and was cleaved for 4 hours at room temperature using a 5 mL of a Met-Reducing cleavage cocktail containing 77.5% TFA, 5% thioanisole, 5% dimethylsulfide, 5% TIPS, 5% TMS-Cl and 2.5% EDT. After 4 hours, the cleavage mixture was filtered, evaporated under N2 flow, and the resulting solid was dissolved in 20 mL of 50% ACN in water + 0.1% formic acid, flash-frozen using liquid nitrogen, and lyophilized.

#### Preparative liquid chromatography-mass spectrometry (Prep LC-MS).

Preparative HPLC purification was performed on an Agilent Technologies mass-directed purification system 1260 Infinity LC coupled to a 6130 Single Quad MS. A Timberline Instrument TL105 HPLC column heater was used to heat the column to 50 °C. The crude lyophilized peptide powder was solubilized in 6 mL of a denaturing buffer consisting of 6 M Gdm HCl, 100 mM TRIS HCl, pH=7.5. The mixture was filtered using a 0.22 *μ*m nylon syringe filter. An analytical HPLC run was first performed using a low volume of protein to estimate %B and a focused gradient was performed for the purification runs as follows:

Analytical run: Column: Agilent Zorbax 300 Stable Bond C3 PrepHT, 21.1 × 100 mm, 5 *μ*m. Flow Rate: 20 mL/min. Solvents: A: H_2_O + 0.1% TFA, B: ACN + 0.1% TFA. Column Temperature: 50 °C. Gradient: 0–10 min 5% B, 10–40 min 5–95% B, 40–45 min 95% B.

Focused gradient run: To generate the final preparative HPLC method, we estimated the %B of elution during the analytical run and performed a preparative run using a linear gradient of −10% to +5%B in 60 minutes around the %B identified in the analytical run. Column: Agilent Zorbax 300 Stable Bond C3 PrepHT, 21.1 × 100 mm, 5 *μ*m. Flow Rate: 20 mL/min. Solvents: A: H_2_O + 0.1% TFA, B: ACN + 0.1% TFA. Column Temperature: 50 °C. Gradient: see description.

Fractions were analyzed by LC-MS, chosen based on apparent purity, flash-frozen using liquid nitrogen and lyophilized.

#### Protein folding via dialysis.

The purified lyophilized fractions were solubilized with a denaturing solution containing 6 M guanidinium chloride, HEPES 10 mM, pH = 7.4 and grouped, using a total volume of less than 6 mL. Solubilized protein was dialysed against a 20 mM TRIS, 200 mM NaCl, pH 7.5 buffer using a 3.5 kDa MWCO dialysis membrane (Slide-A-Lyzer^®^ MINI Dialysis Devices, 2 mL, cat. no. 88403) from Thermo. Buffer changes were performed after 2 and 24 hours for a total of three dialysis cycles over 48 hours.

#### Liquid chromatography-mass spectrometry (LC-MS).

Mass analysis was performed on an Agilent Technologies 1290 Infinity II UHPLC coupled to a 6550 iFunnel Q-TOF LC-MS system. MS spectra were acquired in positive ionization mode with m/z range of 300–2000 Da.:

C4–1-91–10min: Phenomenex Aeris C4 column (2.1 × 150 mm, 3.6 *μ*m, 0.2 mL/min, 40 °C), 1% B for 0–1 min, 1–91% B over 1–7 min, 1% B for 7–9 min. (ESI+, mass range 100–1700 m/z). Column: Phenomenex Aeris WIDEPORE C4 200 Å (2.1 × 150 mm, 3.6 *μ*m, Cat. no. 00F-4486-AN.). Flow Rate: 0.3 mL/min. Solvents: A: H_2_O + 0.1% FA, B: ACN + 0.1% FA. Column Temperature: 40 °C. Gradient: 0–2 min 1% B, 2–8 min 1%–91% B, 8–10 min 91%–95% B (MS acquisition from 2 to 8 min).

Data were processed using Agilent MassHunter BioConfirm software version 10.0. Mass deconvolution was carried out using the 600–2000 m/z mass range and output deconvoluted masses were fixed between 10,000–20,000 Da.

#### Protein concentration.

After three cycles of dialysis (48 hours total), the protein solution was concentrated using pre-humidified 3 kDa MWCO centrifugal filters (Amicon^®^ Ultra – 4). Samples were deposited onto the filters and spun at 4,000 RPM for 99 minutes, or until the combined remaining solution volume was under 500 μL. The resulting solution was taken up into protein lo-bind Eppendorf tubes and assayed for protein concentration (A280) using a Tecan Spark^®^ plate reader. Extinction coefficients were estimated using the ProtParam tool from ExPASy Swiss Institute of Bioinformatics - Bioinformatics Resource Portal using the primary sequence of the protein candidates and specifying C-terminal amide and reduced cysteines to estimate the concentration of the proteins [153].

#### Analytical high-performance liquid chromatography (HPLC).

Analytical HPLC was carried out on an Agilent 1290 series system with UV detection at 214 nm.

C3–5-65–60min: Zorbax 300-SB C3 column (2.1 × 150 mm, 3.6 *μ*m, 0.2 mL/min, 40 °C), 5% B for 0–5 min, 5–65% B over 5–65 min, 65–95% B for 65–66 min, 95% B for 66–70 min, 95–5% B for 70–75 min. Column: Zorbax 300-StableBond C3 (2.1 × 150 mm, 5 *μ*m, cat. no. 883750–909.) Flow Rate: 1 mL/min. Solvents: A: H_2_O + 0.1% FA, B: ACN + 0.1% FA. Column Temperature: 40 °C. Gradient: 0–5 min 5% B, 5–65 min 5%–65% B, 65–66 min 65%–95% B, 66–70 min 95% B, 70–75 min 95–5% B.

UV 214 nm integrations were performed automatically by the built-in Agilent OpenLab CDS, ChemStation Edition Rev.C.01.10[287].

#### Holoprotein reconstitution.

To reconstitute the apo protein, a stock solution of hemin in DMF (concentration variable, 33.3x the concentration of the protein in solution) was added to the protein in buffer in equimolar amounts (1 eq.) with a final concentration of 3% DMF. The solution was vortexed and incubated at room temperature for 5 minutes to allow binding as previously described [154].

#### UV-Vis spectroscopy heme binding assay.

##### Nanodrop methodology (Synthetic candidates):

2 *μ*L of the reconstituted holoprotein solution was deposited into a nanodrop plate (NanoQuant plate) and assayed for a Soret peak at ~450 nm using a Tecan Spark^®^ plate reader at wavelengths between 300 and 650 nm (1 nm step, 3.5 nm slit). All samples were subtracted from a blank containing 3% DMF in buffer, and a negative control using Hemin alone and Hemin + bovine serum albumin (BSA) was performed. Presence of a Soret peak (Soret $\lambda_{\text{max}}$) around 420–430 nm indicated positive heme binding [154, 155]. 384-well plate methodology (Recombinant candidates): Since the concentration of most recombinant candidates was lower than the detection threshold for nanodrop quantification, 120 μL of the reconstituted proteins were added to a 384-well plate from Greiner Bio-One (MICROPLATE, 384 WELL, PS, *μ*CLEAR^®^, WHITE, NON-BINDING, cat. no. 781903). Absorbances were assayed for a Soret peak at ~450 nm using a Tecan Tecan Spark^®^ plate reader at wavelengths between 300 and 650 nm (1 nm step, 3.5 nm slit). All samples were subtracted from a blank containing 3% DMF in buffer, and a negative control using Hemin alone and Hemin + BSA was performed. Presence of a Soret peak (Soret $\lambda_{\text{max}}$) in the range of 420–430 nm indicated positive heme binding [154, 155].

## Supplementary Material

Supplement 1

## Data Availability

Datasets are available on Harvard Dataverse at https://doi.org/10.7910/DVN/4DUBJX.

## Code availability.

The code to reproduce the results, documentation and usage examples is available on GitHub at https://github.com/mims-harvard/ATOMICA. The model weights are available at https://huggingface.co/ada-f/ATOMICA. Details are also available at https://zitniklab.hms.harvard.edu/projects/ATOMICA.
