# Supplementary material for: Learning Universal Representations of Intermolecular Interactions with ATOMICA
Source: bioRxiv. 2025 Jul 15:2025.04.02.646906. Originally published 2025 Apr 8. Preprint. [Version 2] doi: 10.1101/2025.04.02.646906 (PMC12026499; doi:10.1101/2025.04.02.646906)
Supplement: Supplement 1 [file media-1.pdf]

Supplementary Information for

Learning Universal Representations of Intermolecular Interactions with ATOMICA

Ada Fang[1,2,3], Michael Desgagné[4], Zaixi Zhang[3], Andrew Zhou[3,5], Joseph Loscalzo[6,7], Bradley L. Pentelute[4,8,9,10], and Marinka Zitnik[2,3,10,11,‡]

[1]Department of Chemistry and Chemical Biology, Harvard University, Cambridge, MA, USA
[2]Kempner Institute for the Study of Natural and Artificial Intelligence, Harvard University, MA, USA
[3]Department of Biomedical Informatics, Harvard Medical School, Boston, MA, USA
[4]Department of Chemistry, Massachusetts Institute of Technology, Cambridge, MA, USA
[5]Program in Health Sciences and Technology, Massachusetts Institute of Technology, Cambridge, USA
[6]Department of Medicine, Harvard Medical School, Boston, MA, USA
[7]Brigham and Women's Hospital, Boston, MA, USA
[8]Koch Institute for Integrative Cancer Research, Massachusetts Institute of Technology, Cambridge, MA, USA
[9]Center for Environmental Health Sciences, Massachusetts Institute of Technology, Cambridge, MA, USA
[10]Broad Institute of MIT and Harvard, Cambridge, MA, USA
[11]Harvard Data Science Initiative, Cambridge, MA, USA

This PDF file includes:

Supplementary Figures S1 to S5
Supplementary Table S1 to S2

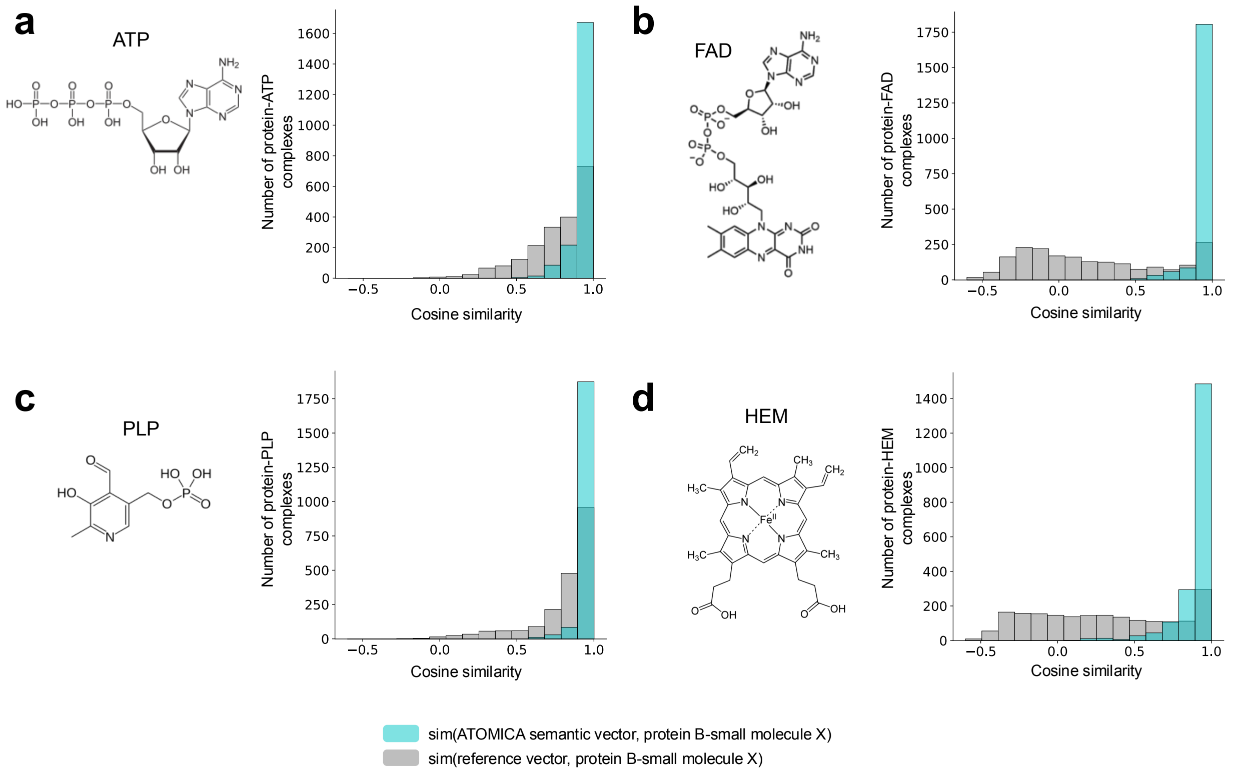

Figure S1: Compositional algebra of protein-small molecule complexes with ATOMICA embeddings We display the cosine similarity of the ATOMICA Semantic Vector with the embedding of protein B-small molecule X. Reference vectors are the embedding of a protein-small molecule complex chosen at random. The following small molecule ligands are shown: **a** ATP, **b** FAD, **c** PLP, and **d** HEM.

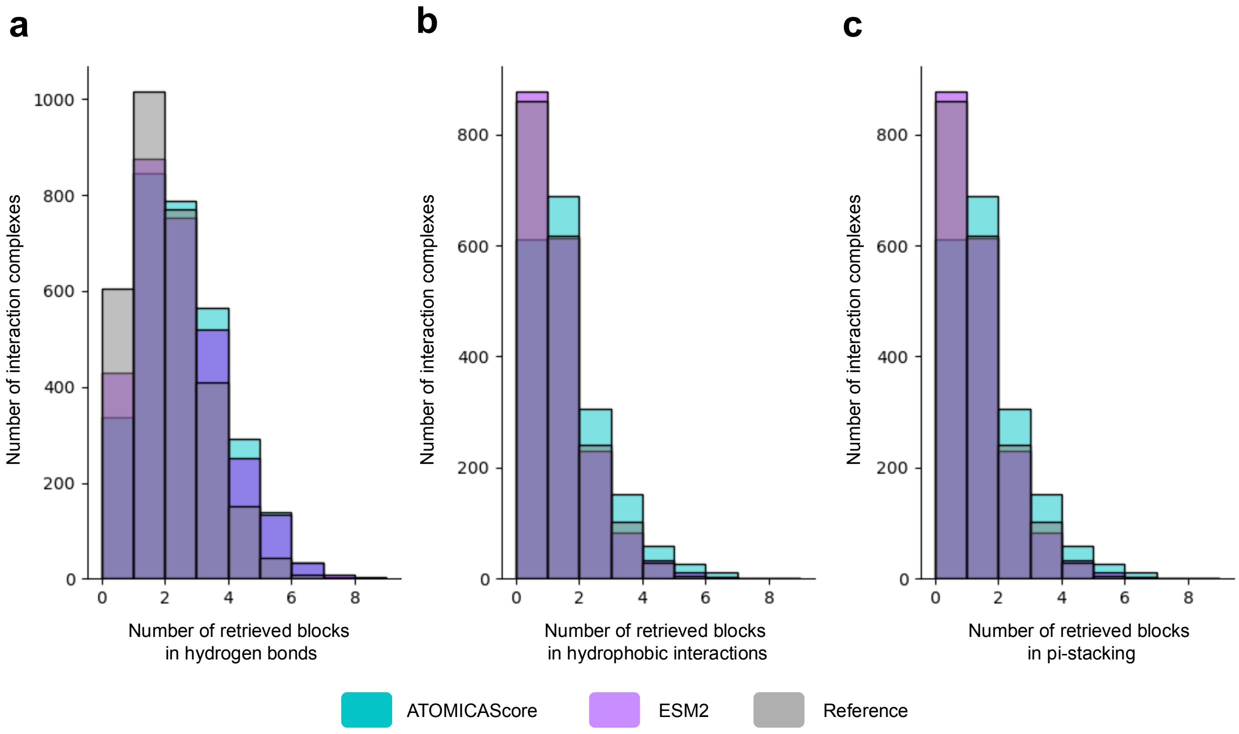

Figure S2: Number of blocks involved in intermolecular bonds in the top 10 nominated blocks for protein-small molecule complexes in the pretraining test set. We compare ATOMICASCORE, ESM-2 (3B), and a reference on the recovery of the following types of intermolecular bonds: **a** hydrogen bonds, **b** hydrophobic interactions, and **c** pi-stacking.

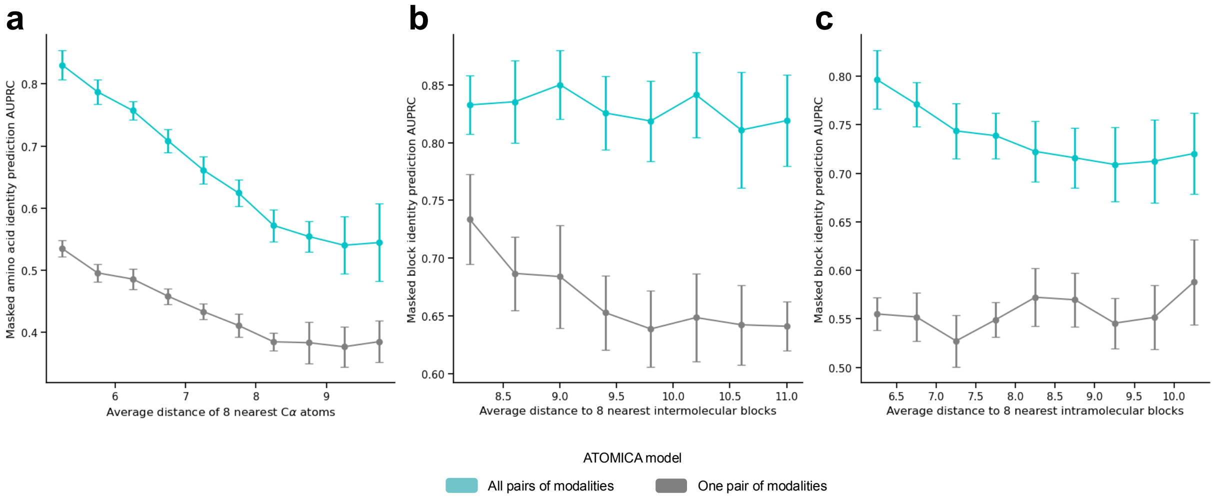

Figure S3: Masked block identity AUPRC of ATOMICA models trained on all pairs of interacting modalities compared to one pair of interacting modalities. **a** Masked amino acid identity prediction AUPRC binned by average distance to 8 nearest Cα atoms. **b** Masked block identity identity prediction AUPRC binned by average distance to 8 nearest intermolecular blocks. **c** Masked block identity identity prediction AUPRC binned by average distance to 8 nearest intramolecular blocks.

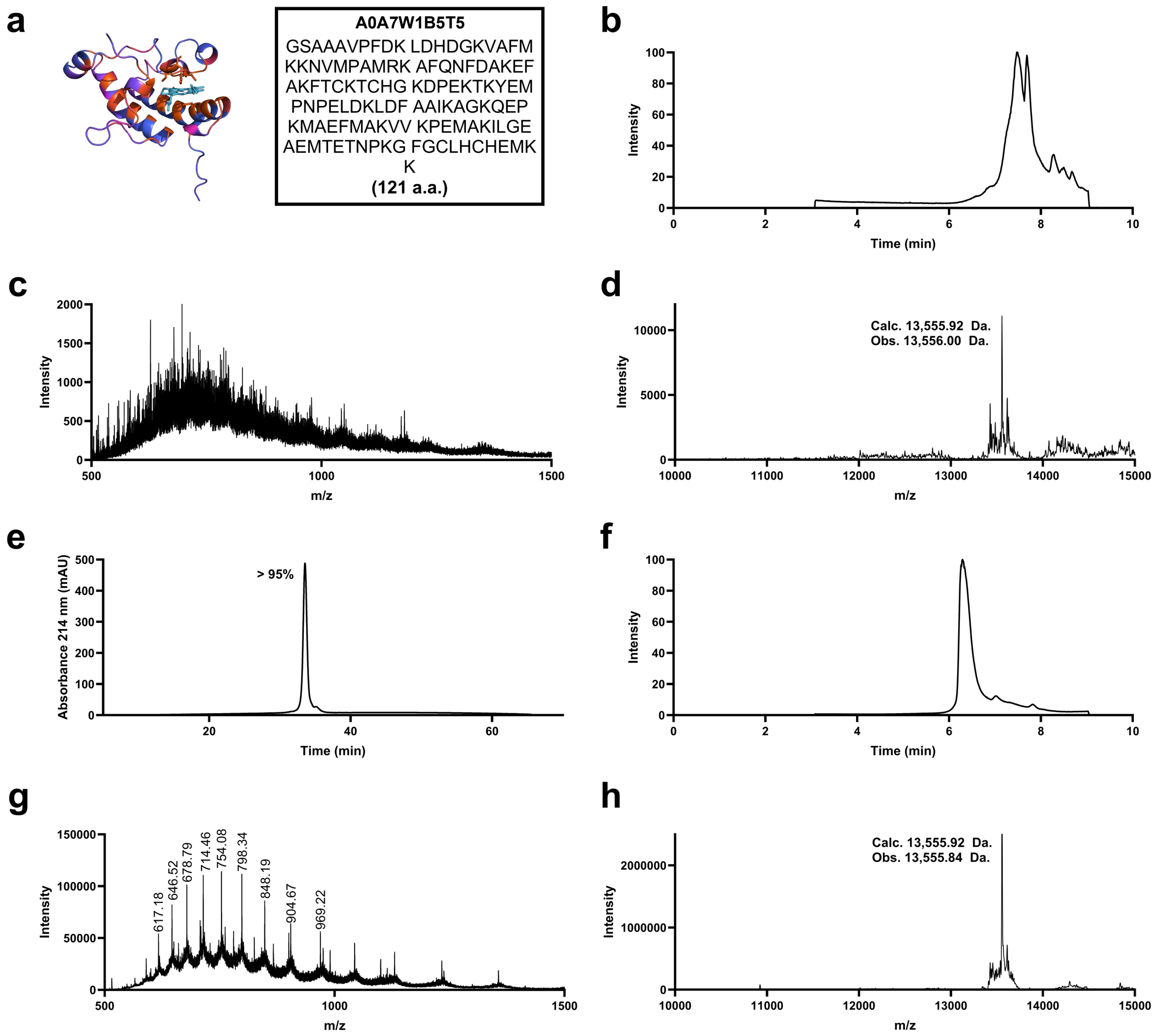

Figure S4: Chemical characterization of AFPS-synthesized A0A7W1B5T5. **a** AlphaFold2 predicted structure of A0A7W1B5T5 and its sequence. **b** Crude LC-MS spectra (Method: C4-1-91-10min). **c** Crude MS TIC scan (7.420-7.552 min, 13 scans). **d** Crude deconvoluted MS spectra. Calculated $[M+H]^+$ = 13,555.92 Da, Observed $[M+H]^+$ = 13,556.00 Da. **e** Purified UHPLC spectra (214 nm, Method: C3-5-65-60min). ¿95% purity by UV integration. **f** Purified LC-MS spectra (Method: C4-1-91-10min). **g** Purified MS TIC scan (6.284-6.339 min, 6 scans). **h** Purified deconvoluted MS spectra of Candidate 1. Calculated $[M+H]^+$ = 13,555.92 Da, Observed $[M+H]^+$ = 13,555.84 Da.

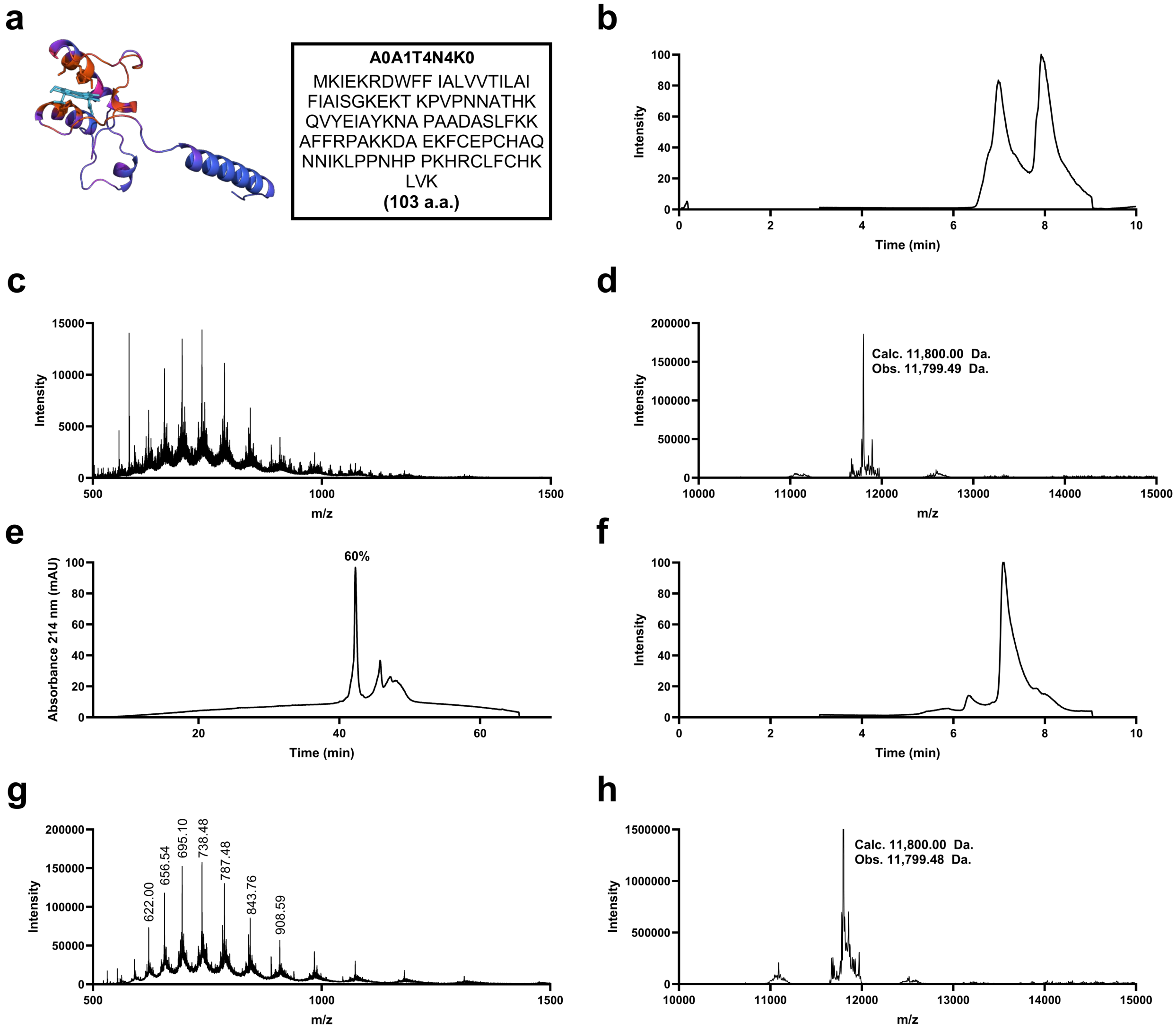

Figure S5: Chemical characterization of AFPS-synthesized A0A1T4N4K0. **a** AlphaFold2 predicted structure of A0A1T4N4K0 and its sequence. **b** Crude LC-MS spectra (Method: C4-1-91-10min). **c** Crude MS TIC scan of (7.878-8.066 min, 18 scans). **d** Crude deconvoluted MS spectra. Calculated $[M+H]^+$ = 11,800.00 Da, Observed $[M+H]^+$ = 11,799.49 Da. **e** Purified UHPLC spectra (214 nm, Method: C3-5-65-60min). 60% purity by UV integration. **f** Purified LC-MS spectra (Method: C4-1-91-10min). **g** Purified MS TIC scan (7.063-7.151 min, 9 scans). **h** Purified deconvoluted MS spectra of Candidate 2. Calculated $[M+H]^+$ = 11,800.00 Da, Observed $[M+H]^+$ = 11,799.48 Da.

Table S1: ATOMICANET disease protein counts. Number of disease proteins across ion, small molecule, lipid, nucleic acid, and protein ATOMICANETs for 82 diseases.

Disease name	Disease ID	Number of disease proteins in ATOMICANET				
		Ion	Small-Molecule	Lipid	Nucleic Acid	Protein
Alzheimer disease	MONDO_0004975	17	25	27	10	43
Charcot-Marie-Tooth disease	MONDO_0015626	24	27	17	24	59
Joubert syndrome	MONDO_0018772	15	13	12	10	37
Leigh syndrome	MONDO_0009723	9	15	16	4	36
Noonan syndrome	MONDO_0018997	20	27	5	14	31
Parkinson disease	MONDO_0005180	27	31	53	21	73
Spinocerebellar ataxia with oculomotor anomaly	Orphanet_98693	22	16	11	15	30
T-cell and NK-cell neoplasm	MONDO_0024615	19	22	5	16	28
adrenal gland disease	EFO_0005539	16	16	36	14	44
amyotrophic lateral sclerosis	MONDO_0004976	15	11	17	18	34
aplastic anemia	MONDO_0015909	26	23	9	37	50
arterial occlusive disease	EFO_0009085	16	15	42	8	68
arteriosclerosis	EFO_0009086	16	15	42	8	68
asthma	MONDO_0004979	16	7	43	16	46
atherosclerosis	EFO_0003914	16	15	42	8	68
autism spectrum disorder	EFO_0003756	19	10	20	10	30
autoimmune neuropathy	MONDO_0000774	39	2	43	11	44
basal ganglia disease	EFO_0009533	32	34	67	24	87
bile duct carcinoma	EFO_0005540	15	16	8	17	27
blood coagulation disease	EFO_0009314	23	28	24	24	51
blood platelet disease	MONDO_0002245	22	15	19	16	31
bone marrow cancer	MONDO_0021138	64	51	23	65	90
breast neoplasm	EFO_0003869	141	145	136	184	345
cardiac arrhythmia	EFO_0004269	25	14	83	25	78
cardiomyopathy	EFO_0000318	75	77	77	52	145
cataract	MONDO_0005129	7	7	18	13	30
cerebellar ataxia	MONDO_0000437	42	36	37	34	66
cerebrovascular disorder	EFO_0003763	42	28	58	15	73
chronic lymphocytic leukemia	EFO_0000095	17	16	5	18	26
colon carcinoma	EFO_1001950	21	15	8	21	31
coronary artery disease	EFO_0001645	49	41	93	22	135
deafness	EFO_0001063	19	13	21	24	47
depressive disorder	MONDO_0002050	26	21	79	23	67
diabetes mellitus	EFO_0000400	65	44	105	52	160
ectodermal dysplasia syndrome	MONDO_0019287	27	26	13	27	56
epilepsy	EFO_0000474	105	100	139	66	220
esophageal disease	EFO_0009544	28	37	29	32	57

familial hemolytic anemia	MONDO_0003689	13	17	11	10	25
glaucoma	MONDO_0005041	14	12	19	11	27
glomerular disease	EFO_1002049	14	13	19	16	37
glycoprotein metabolism disease	MONDO_0045010	10	20	25	11	33
head and neck squamous cell carcinoma	EFO_0000181	17	31	9	22	30
hepatocellular carcinoma	EFO_0000182	13	15	14	17	32
hereditary dementia	MONDO_0015547	31	34	41	23	65
hereditary spastic paraplegia	MONDO_0019064	28	29	33	15	55
hypertrophic cardiomyopathy	EFO_0000538	42	45	23	30	77
hypogonadism	MONDO_0002146	20	16	27	33	48
hypotension	EFO_0005251	6	8	34	5	31
inborn carbohydrate metabolic disorder	MONDO_0019214	60	76	42	29	116
inborn disorder of amino acid metabolism	MONDO_0004736	87	93	77	43	180
inborn disorder of purine or pyrimidine metabolism	MONDO_0019254	25	25	8	10	38
inflammatory bowel disease	EFO_0003767	29	24	31	22	42
inherited lipid metabolism disorder	MONDO_0002525	38	78	92	16	114
lymphoma	EFO_0000574	107	102	72	123	204
lysosomal storage disease	MONDO_0002561	25	38	35	10	68
macular degeneration	EFO_0009606	6	7	16	13	18
malignant glioma	MONDO_0100342	11	19	11	23	31
mitochondrial complex I deficiency	MONDO_0100133	7	8	14	4	38
motor neuron disease	EFO_0003782	29	32	32	39	83
multiple myeloma	EFO_0001378	18	24	15	10	32
multiple sclerosis	MONDO_0005301	61	25	90	30	103
myelodysplastic syndrome	EFO_0000198	16	16	6	18	29
myeloid leukemia	MONDO_0004643	53	48	20	58	68
myeloproliferative disorder	EFO_0004251	25	19	11	24	36
neuroendocrine carcinoma	MONDO_0002120	11	20	14	24	25
non-small cell lung carcinoma	EFO_0003060	39	63	34	57	84
normocytic anemia	MONDO_0004139	18	21	16	16	32
osteoarthritis	MONDO_0005178	16	11	48	17	40
pancreatic carcinoma	EFO_0002618	24	36	11	24	42
peroxisomal disease	MONDO_0019053	11	12	18	7	26
polycystic ovary syndrome	EFO_0000660	14	9	36	13	60
prostate cancer	MONDO_0008315	36	46	35	42	67
psoriasis	EFO_0000676	17	9	17	15	29

renal cell carcinoma	EFO_0000681	15	21	16	25	40
renal tubule disease	EFO_0009566	37	28	37	23	62
rheumatoid arthritis	EFO_0000685	15	15	21	10	29
sarcoma	EFO_0000691	32	48	26	45	70
schizophrenia	MONDO_0005090	26	28	105	26	117
spinal muscular atrophy	EFO_0008525	6	11	10	12	29
spondyloepiphyseal dysplasia	MONDO_0016761	9	12	10	8	21
thyroid cancer	MONDO_0002108	18	33	25	31	41
vesiculobullous skin disease	EFO_1000774	13	11	13	14	22

Table S2: ATOMICA-Ligand predicted heme-binding experimental candidates. Concentration and apparent purities of all selected ATOMICA-Ligand predicted heme-binding candidates produced by both AFPS and recombinant methodologies.

Protein	Production method	Calc. M.W. (Da)	Conc. ($mg\,mL^{-1}$)	Conc. (μM)	Apparent purity (%)
A0A7W1B5T5	AFPS	14 728	3.23	238.50	$> 95^{\#}$
A0A1T4N4K0	AFPS	12 841	1.39	117.00	$60^{\#}$
A0A7W1B5T5	Recombinant	14 728	0.39	26.48	$\geq 90^{*}$
A0A1T4N4K0	Recombinant	12 841	0.06	4.67	n.d.†
A0A4P5TA35	Recombinant	18 043	0.10	5.54	n.d.†
A0A7W0X6V6	Recombinant	9 115	0.25	27.43	$\geq 70^{*}$
A0A2V6P8N7	Recombinant	8 576	0.10	11.66	n.d.†
V5BF69	Recombinant	70 349	0.09	1.28	$\geq 80^{*}$
A0A136KY61	Recombinant	24 261	0.32	13.19	$\geq 95^{*}$
A0A7Y8LED7	Recombinant	58 116	0.08	1.38	$\geq 75^{*}$
A0A7V7N0X5	Recombinant	51 703	0.06	1.16	n.d.†
BSA control	Commercial	66 430	1.49	22.46	n.d.

$*$: Assayed by GenScript using SDS-PAGE. #: Assayed using $A_{214\,nm}$ by UHPLC.

†: Protein quantity too low to assay purity via SDS-PAGE. n.d. = not determined.
